# Extensive transcriptional responses are co-ordinated by microRNAs as revealed by Exon–Intron Split Analysis (EISA)

**DOI:** 10.1093/nar/gkz664

**Published:** 2019-08-02

**Authors:** Katherine A Pillman, Kaitlin G Scheer, Emily Hackett-Jones, Klay Saunders, Andrew G Bert, John Toubia, Holly J Whitfield, Sunil Sapkota, Laura Sourdin, Hoang Pham, Thuc D Le, Joseph Cursons, Melissa J Davis, Philip A Gregory, Gregory J Goodall, Cameron P Bracken

**Affiliations:** 1 Centre for Cancer Biology, an alliance of SA Pathology and University of South Australia, Adelaide, SA, Australia; 2 ACRF Cancer Genomics Facility, Centre for Cancer Biology, SA Pathology, Adelaide, Australia; 3 Bioinformatics Division, Walter and Eliza Hall Institute of Medical Research, Parkville, Victoria, Australia; 4 School of Information Technology and Mathematical Sciences, University of South Australia, Mawson Lakes, SA, Australia; 5 Department of Medical Biology, Faculty of Medicine, Dentistry and Health Sciences, University of Melbourne, Parkville, Victoria, Australia; 6 Department of Biochemistry, Faculty of Medicine, Dentistry and Health Sciences, University of Melbourne, Parkville, Victoria, Australia; 7 School of Medicine, Discipline of Medicine, University of Adelaide, SA, Australia

## Abstract

Epithelial–mesenchymal transition (EMT) has been a subject of intense scrutiny as it facilitates metastasis and alters drug sensitivity. Although EMT-regulatory roles for numerous miRNAs and transcription factors are known, their functions can be difficult to disentangle, in part due to the difficulty in identifying direct miRNA targets from complex datasets and in deciding how to incorporate ‘indirect’ miRNA effects that may, or may not, represent biologically relevant information. To better understand how miRNAs exert effects throughout the transcriptome during EMT, we employed Exon–Intron Split Analysis (EISA), a bioinformatic technique that separates transcriptional and post-transcriptional effects through the separate analysis of RNA-Seq reads mapping to exons and introns. We find that in response to the manipulation of miRNAs, a major effect on gene expression is transcriptional. We also find extensive co-ordination of transcriptional and post-transcriptional regulatory mechanisms during both EMT and mesenchymal to epithelial transition (MET) in response to TGF-β or miR-200c respectively. The prominent transcriptional influence of miRNAs was also observed in other datasets where miRNA levels were perturbed. This work cautions against a narrow approach that is limited to the analysis of direct targets, and demonstrates the utility of EISA to examine complex regulatory networks involving both transcriptional and post-transcriptional mechanisms.

## INTRODUCTION

MicroRNAs confer robustness to biological systems, buffering against stochastic fluctuations and transcriptional noise (1–4), fine-tuning gene expression and acting as agents to promote phenotypic switching between mutually exclusive cell states (5). Fundamental to these functions are the close interplay with transcription factors (TFs), with which they form network motifs that integrate transcriptional and post-transcriptional signals, such as feedback loops (where the expression of a miRNA and a TF are directly dependent upon one another) and feedforward loops (where at least one input TF or miRNA regulates the other and where both jointly regulate shared target genes) (6–8). Such motifs are over-represented within the architectures of mammalian regulatory networks (9–11). Due to the importance and abundance of feedforward and feedback motifs, several databases and predictive tools have been developed to identify them (8,12–17), with each predicting TF:miRNA:target interactions, then matching these predictions to gene expression changes within the transcriptomic milieu of cells. However, the many levels of gene regulation obscure the relative importance of the transcriptional and post-transcriptional mechanisms—and thus the direct and indirect roles played by individual TFs and miRNAs.

One biologically important process that is controlled by complex interactions between TFs and miRNAs is epithelial–mesenchymal transition (EMT), a reversible phenotypic switch that underlies normal processes such as gastrulation, neural crest delamination and wound healing, and pathologies, including fibrosis, metastasis and chemoresistance (18). Under the influence of multiple signalling pathways, EMT-inducing TFs (including members of the Zeb, Snail and Twist families) directly or indirectly repress epithelial genes such as E-cadherin which is a hallmark of the epithelial phenotype. The expression of these TFs are controlled through feedback interactions with miRNAs such as those of the miR-200 and miR-34 families, whose expression both facilitate stable epithelial or mesenchymal phenotypes, and permit the existence of a partial EMT state that is hypothesised to play a role in metastasis (19–21).

In order to uncover the roles played by miRNAs and miRNA:TF regulatory loops in EMT, we used a bioinformatics technique known as EISA (Exon–Intron Split Analysis), which delineates transcriptional and post-transcriptional regulation of mRNA through the separate analysis of sequencing reads mapping to exons and introns (22). Changes in the abundance of intron-mapping reads (ΔI) reflect altered rates of transcription, whilst the difference in the abundance of reads mapping to exons minus the abundance of reads mapping to introns (ΔE – ΔI) reflects the post-transcriptional regulation of mRNA stability, much of which is due to miRNAs and is further augmented at the level of translation.

By using EISA to help identify direct post-transcriptional targets of miR-200c, an epithelial-promoting miRNA, we found (and verified) an over-representation of transcripts whose products act downstream of the Epidermal Growth Factor Receptor (EGFR), implying co-ordinated suppression of this mesenchymal-promoting signalling pathway. This however only represented a small portion of miR-200c responsive genes, with the majority being transcriptionally upregulated, most likely a downstream effect of miR-200c directly targeting transcriptional repressors such as, but not limited to, ZEB family members. Importantly, transcriptionally upregulated genes were highly enriched for functionality in EMT-associated processes such as maintenance of cell-cell junctions, cell adhesion and extracellular matrix organisation, suggesting that the enactment of biological effects by miRNAs includes a significant, and often overlooked, contribution by indirect targets at the transcriptional level. We find extensive transcriptional effects are observed for other miRNAs, suggesting that widespread, co-ordinated regulation of biological processes across different gene regulatory layers is common, despite transcription and miRNA-directed mRNA stability being physically de-coupled as they occur in separate cellular compartments. More generally, this work highlights the need to consider the ‘indirect’ effects of miRNAs on transcription that the examination of direct targets alone fail to capture and supports EISA as a useful tool to help delineate such gene regulatory processes.

## MATERIALS AND METHODS

### Cell culture and transfection

HMLE cells (23) were cultured in HuMEC Ready Media (ThermoFisher) and induced to undergo EMT by transferring to DMEM:F12 media (1:1) supplemented with 10 lg/ml insulin, 20 ng/ml EGF, 0.5 lg/ml hydrocortisone and 5% fetal calf serum (FCS) and treating with 2.5 ng/ml of TGF-β1 (R&D) for at least 14 days. MesHMLE cells, which are derived from HMLE from prolonged treatment with TGF-β1, were maintained in EMT-inducing media without additional TGF-β1. MDCK cells were grown in DMEM + 10% FCS. MesHMLE cells were reverted toward an epithelial state by transfection with 20 nM miR-200c precursor (mirVana miRNA mimics; Ambion) for 72 h using Lipofectamine RNAiMAX (ThermoFisher). miR-200c antagomiRs (100nM, Ambion) were transfected into HMLE cells for 72 h and into MDCK cells every 3 days for a total of 9 days prior to lysis.

### Luciferase assay

Target 3′UTRs were cloned downstream of Renilla luciferase in the pRL vector (Promega). Assays were performed co-transfecting 10 ng RL construct, 10 ng of the pGL3 (Promega) internal control and 10 nM miR-200c (or matched negative control) into wells (24-well tray) containing ∼35 000 cells using Lipofectamine 2000 (ThermoFisher). Twenty four hours post-transfection, cells were lysed and luciferase activity determined using the Twin Luciferase Reporter Assay kit (Biotool). A ratio of Renilla/Firefly luminescence intensity was used to calculate the relative luciferase expression activity.

### EGF stimulation and western blotting

Cells were transfected with 10 nM control or miR-200c mimics as previously described for 72 h, then starved in unsupplemented DMEM:F12 media for 6 hours prior to stimulation with 20 ng/ml EGF for 15 min. Cells were then lysed in RIPA buffer (Abcam) to which protease and phosphatase inhibitor cocktails (Roche) were added. Proteins were separated by electrophoresis, transferred to nitrocellulose and blocked with odyssey blocking buffer for 1 h prior to overnight incubation with the following antibodies (at 1:1000 dilution): MEK1/2 (CSL, #8727), pMEK1/2 (CSL, #9154S), ERK1/2 (CSL, #4696S), pERK1/2 (CSL, #9101S), AKT (CSL, #2920S), pAKT (CSL, #4056SO, EGFR (CSL, #4267S), pEGFR (CSL, #4407S) and a-tubulin (Santa Cruz, #E0415). Blots were then washed 3 × 5 min with TBS-Tween and incubated with secondary antibodies (1:20 000 dilution): Goat anti-rabbit 680 (red) (Millennium, #926-68071), Goat anti-mouse 800 (green) (Millennium, #926-32210), Goat anti-rabbit 800 (green) (Millennium, #926-32211).

### Chromatin modification ChIP analysis

ChIP assays were performed as described in, except for the use of a probe sonicator instead of a BioRupror. Probe was set to 35% intensity for 20 × 30 s sonication/rest cycles. Antibodies used are as follows: H3K4me3 (Abcam, #ab8580), H3K9/14ac (Millipore, #06-599), H3K27ac (Millipore, #17-683), H3K27me3 (Abcam, #ab6002).

### Read processing and alignment

For EISA analysis, raw reads were adapter trimmed and filtered using cutadapt v1.8 (24), and for the resulting FASTQ files quality checks were performed with FastQC(https://www.bioinformatics.babraham.ac.uk/projects/fastqc/). Filtered reads were mapped against the human reference genome (build hg19) using the STAR (v2.5.3a) spliced alignment algorithm (25) with default parameters. Alignments were visualized and interrogated using the Integrative Genomics Viewer v2.3.80 (26).

### Exon–Intron Split Analysis

EISA analysis was performed essentially as described in (22). Briefly, we used only non-overlapping genes and uniquely mapped reads to quantify the number of reads in intronic and exonic regions in a strand-specific manner for all UCSC RefSeq mRNA transcripts from each gene. Read pairs were ‘exonic’ if the 5′ end of the first read was mapped within an exon of any UCSC transcript or ‘intronic’ if the first read mapped entirely within an intron and not within 10 bp of an exon. The custom python script used to obtain counts of reads mapping to introns and exons was based on the pyreference package (https://bitbucket.org/sacgf/pyreference), and is described in (27), the script ‘cursons_bam_get_eisa_counts.py’ and usage instructions are available from the associated Github repository https://github.com/DavisLaboratory/Combinatorial_miRNAs). Statistical analysis of read counts was performed using R scripts based on those in (22), using the EdgeR (28) and limma (29).

### Post-EISA analyses

To describe the transcriptional/post-transcriptional effect of an experimental condition we devised the following metric to measure the relative contribution of each component. We considered each gene on the axes (*x*,*y*) = (ΔI, ΔE – ΔI). Genes were thresholded by (*x* + *y* > 1) to include only differentially expressed genes in the metric. The post-transcriptional contribution to differential expression is defined as the fraction of these genes with absolute post-transcriptional change abs (ΔE – ΔI) greater than the absolute transcriptional change abs(ΔI). Conversely, the transcriptional contribution is the fraction of these genes with absolute transcriptional change abs(VI) greater than the absolute transcriptional change abs (ΔE – ΔI).

### Sequence read downsampling analysis

Raw data were processed as outlined above however, prior to mapping, samples were down-sampled to seven different depths to simulate various sequencing depths. Six HMLE and four mesHMLE samples all containing more than 60 million reads were down-sampled to 60, 50, 40, 30, 20, 10 and 5 million reads. Down-sampled samples were then aligned and EISA performed as above.

### Datasets

The datasets for miR-200c transfection of MesHMLE cells with TGF-β were prepared previously as described in (30) and (31), respectively and the raw data obtained from the European Nucleotide Archive under the accessions provided in Supplementary Table S1. For public datasets, raw fastq files were downloaded from repositories using the accessions supplied in Supplementary Table S1. Transcription factor target genes obtained from MACS peak calling of ChIP-Seq data (Figure 3E) were obtained from the Gene Transcription and Regulation Database (GTRD) (32). The enrichment of binding sites among genes that are transcriptionally upregulated after miR-200c expression (Figure 3C) was determined using the cytoscape plug-in, iRegulon (33,34), which calculates enrichment scores among 181 different transcription factors assessed by the ENCODE consortium (34).

### Histone mark ChIP-seq data processing

ChIP-seq libraries were prepared for trimethyl histone H3K4 (ab8580; Abcam), acetyl histone H3K9/14, acetyl histone H3K27 and trimethyl histone H3K27 and 10% input samples for both HMLE and MesHMLE cells as described in (31). Peak calling was performed using MACS (v1.4.2) (35) using sizeshift parameters of between 150 and 163 (equal to half the average fragment size as determined using Bioanalyzer), setting the nomodel parameter and otherwise default parameters. Alignment files and MACS peak files were converted to bed format and used as input for MAnorm (R version) (36) which normalised peak data and determined differential peak enrichment (MA norm ‘M’ values) between HMLE and MesHMLE samples for each histone modification mark (supplying the average fragment size for each sample as described for above). The peak-centric MAnorm output was converted to gene-centric format as follows: for each gene, an *M*-value was calculated from the peaks overlapping the promoter (1kb up- and down-stream of the transcriptional start site) as: 0 if no peaks overlapped the promoter, if one peak overlaps, then the *M*-value of that peak was used, if two or more peaks overlap then the *M*-value of the largest peak (i.e. with the greatest *A*-value) was used. The resulting *M*-value for each gene represented the log_2_ fold-change in histone modification in the promoter of that gene between HMLE and MesHMLE cells and was plotted on the *y*-axis (‘relative enrichment’) for the ChIP-seq figures.

## RESULTS

### EISA successfully differentiates transcriptional and post-transcriptional gene regulation

**Figure 1.**
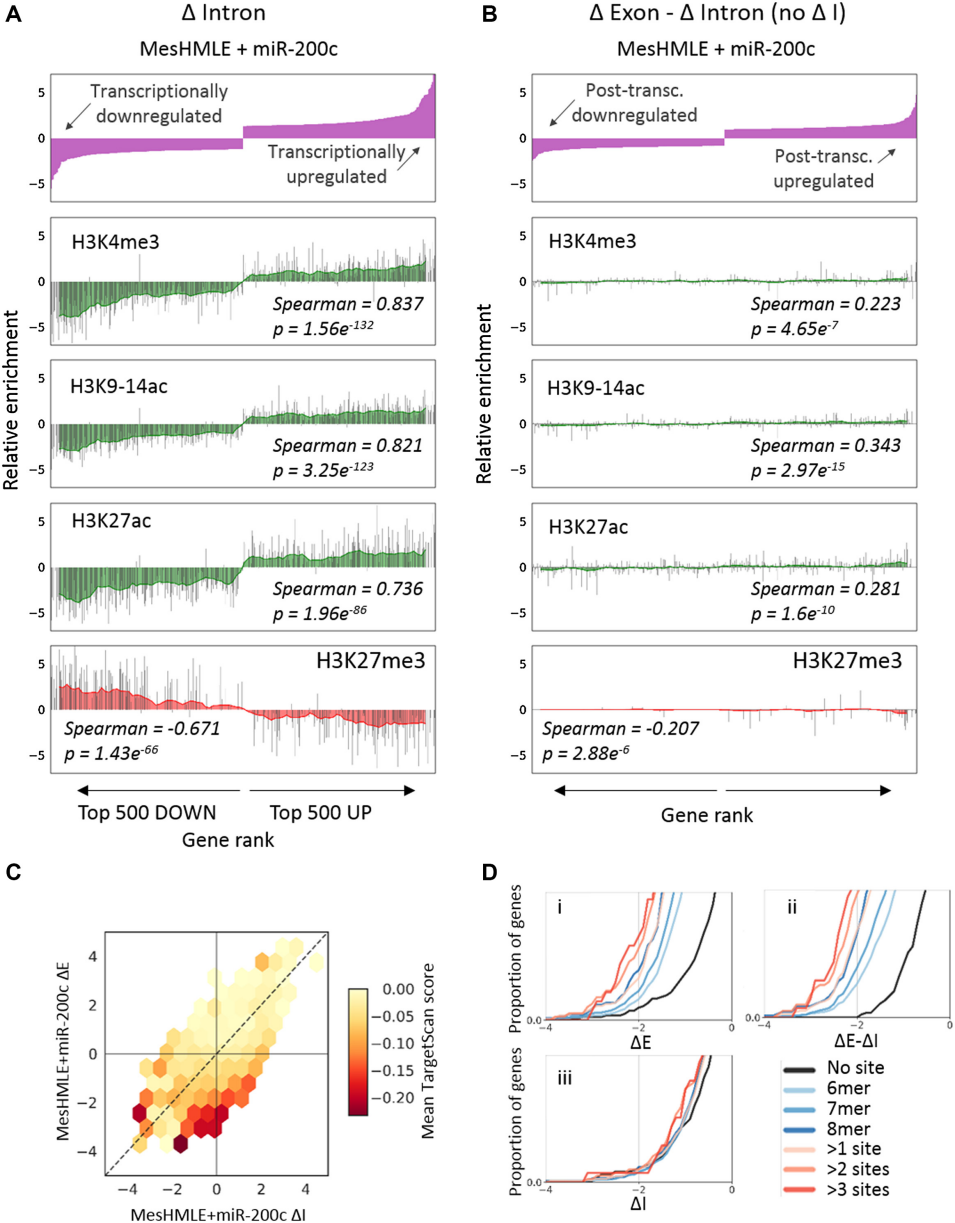
EISA effectively delineates transcriptional and post-transcriptional gene regulation. (**A**) The top 5% up- and down-regulated genes were ranked according to the degree of transcriptional (ΔI) regulation after miR-200c-driven mesenchymal to epithelial transition. The relative enrichment of markers of active (H3K4me3, H3K9-14ac, H3K27ac – green) and inactive (H3K27me3 – red) chromatin from ChIP-Seq is shown for each gene in the plot. The red/green shading shows the mean of these measurements in a sliding window across 25 genes (plotting every 5th window). (**B**) As for (A), except that genes are ranked according to ΔE – ΔI, and only for genes with little evidence of transcriptional regulation (ΔI <0.5). (**C**) EISA was used to plot genes that are responsive to miR-200c on a ΔI:ΔE axis. Genes are coloured according to the strength of their direct miR-200c target (TargetScan) prediction; the deeper the red colour, the stronger the prediction. (**D**) Cumulative distribution of gene expression fold changes in response to miR-200c expression in MesHMLE cells as determined by (i) ΔExon, (ii) ΔExon-ΔIntron and (iii) ΔIntron (for genes with small ΔE – ΔI <0.5), genes are subcategorised according to whether they are predicted to contain miR-200c target site(s) of the specified length (6-8mers) and number (0 to >3 sites). Pairwise Kolmogorov–Smirnov (K–S) statistical tests (Supplementary Table S2) demonstrate that genes possessing longer or more target sites are progressively more repressed after miR-200c expression when assessed by ΔExon or ΔExon-ΔIntron, but not ΔIntron. EISA (ΔExon – ΔIntron) further enhances statistical significance compared to RNA-Seq (ΔExon).

In an effort to better understand the contributions of miRNAs and miRNA-TF regulatory loops within EMT, we first sought to evaluate the capacity of EISA to differentiate between gene regulatory mechanisms within RNA-sequencing data. To do so, we initially compared concordance between the change in abundance of intronic reads (ΔI) with markers of active and inactive genes in human mammary epithelial cells (HMLEs), a cell model of EMT that can be driven toward a mesenchymal state (MesHMLE) through TGFβ exposure (Figure 1A). All histone marks were strongly correlated with the expression of genes that were indicated by EISA to be transcriptionally regulated (large ΔI). Conversely, markers of active and inactive genes showed little correlation with genes that were regulated post-transcriptionally (large ΔE – ΔI, minimal ΔI, Figure 1B). Thus the EISA metric ΔI reliably indicates effects on gene transcription.

EISA can also aid the identification of direct miRNA targets from RNA-Seq by enriching for transcripts subject to post-transcriptional regulation (22,37). Because the miR-200 family are prominent regulators of EMT and enforcers of the epithelial phenotype (38–42), we applied EISA analysis to RNA-seq data from mesHMLE cells transfected with miR-200c to evaluate the relative transcriptional and post-transcriptional effects. Plotting ΔE versus ΔI, we observed an enrichment in strongly predicted miR-200c targets for the most negative values of the post-transcriptional measure, ΔE – ΔI (observed as deviation from the ΔE = ΔI line) (Figure 1C). Furthermore, excluding transcriptional changes (by using ΔE – ΔI) better delineated the impact of predicted target binding site quality (seed match length and number of binding sites) than when transcriptional changes were not excluded (Figure 1Di,ii, Supplementary Table S2). As a control, we determined that for genes that were indicated by EISA to be solely regulated transcriptionally (Large ΔI, ΔE – ΔI <0.5, equivalent to approximately the least changeable 20% of genes) expression changes were unrelated to quality or number of predicted 3′UTR target sites (Figure 1D,iii). Collectively, these data demonstrate that EISA successfully differentiates between transcriptional and post-transcriptional gene regulation.

To provide an indication of the sequencing depth required for EISA to be a useful adjunct to analysis, we examined the impact of sequencing depth by performing a down-sampling analysis on this dataset. For most datasets, the statistical power is limited by sequencing depth in intronic rather than exonic regions. Furthermore, we have found intron-mapping proportions vary between experiments and sample-preparation methods, making intronic read counts a more useful indicator than total read counts of the suitability and performance of a dataset for EISA. This analysis revealed the number of genes that can be determined as significantly differentially expressed via transcriptional or post-transcriptional regulation for a range of intron-mapping depths (Supplementary Table S3).

### miR-200c post-transcriptionally regulates the EGF signalling network

**Figure 2.**
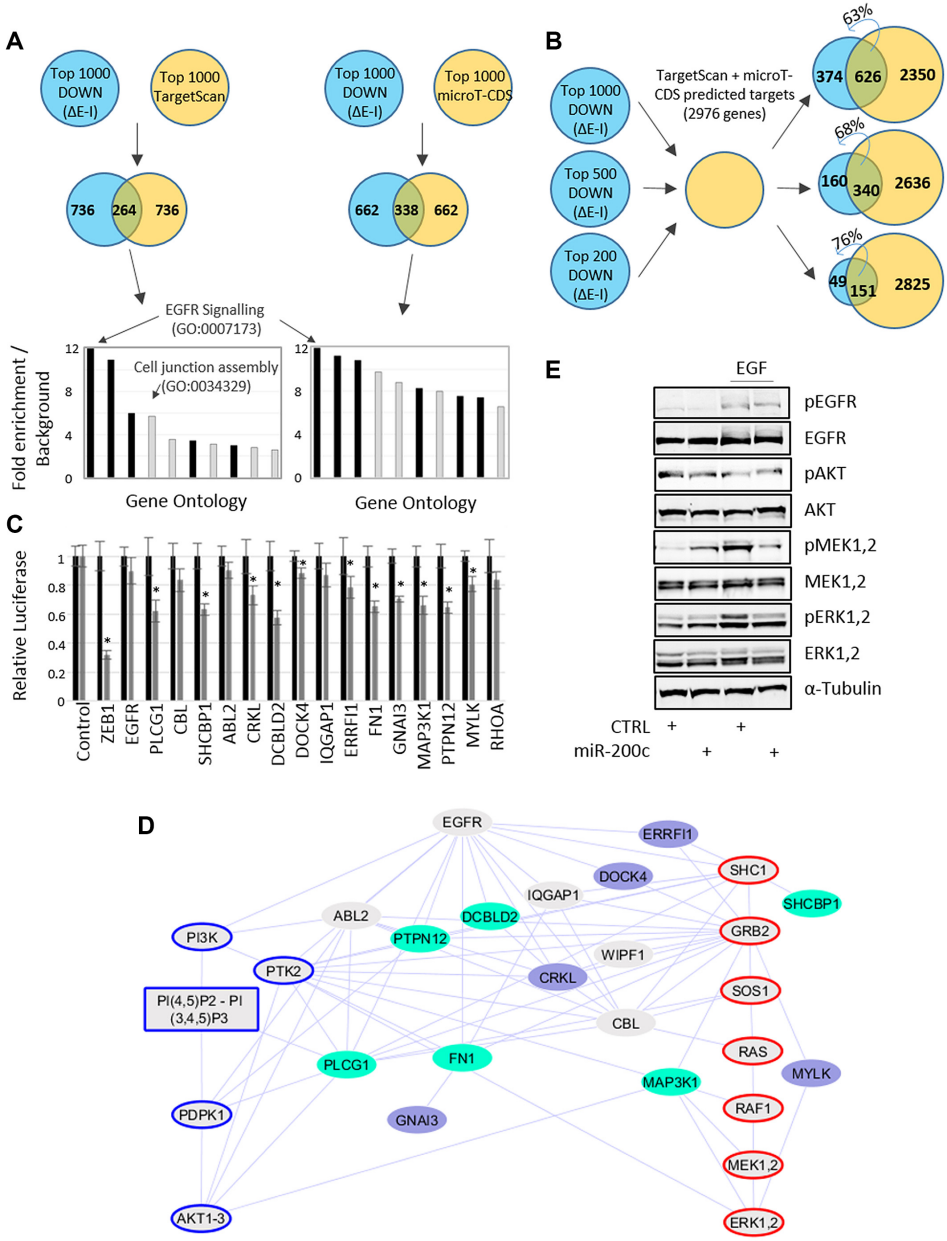
miR-200c directly co-ordinately regulates an EGF signalling network. (**A**) Putative direct miR-200c target genes were defined as genes in the top 1000 of all negatively post-transcriptionally regulated genes (ΔE – ΔI) that are also in the top 1000 of all predicted miR-200c targets (by TargetScan or microT-CDS). The 10 highest fold enrichments over background for the top ranking gene ontologies are indicated. Black columns represent alternate entries for the EGFR signalling pathway within the GoPanther Gene Ontology database. (**B**) Overlap between the 1000, 500 and 200 most post-transcriptionally downregulated genes in response to miR-200c and all miR-200c targets that are predicted by both the targetscan and microT-CDS algorithms. (**C**) 3′UTR-luciferase reporter assays for representative putative miR-200c targets within the EGF signalling network indicate multi-component targeting. Black bars indicate transfection of a control miRNA sequence. Gray bars indicate miR-200c co-transfection. Error bars represent standard deviation, * denote significant (*P*< 0.05) downregulation of the reporter in response to miR-200c as calculated by *t*-test. (**D**) Network representation of miR-200c target genes (shaded blue) within the signalling network downstream of the EGFR. Major signalling pathways leading to AKT (blue outline) and ERK (red outline) activation are adapted from KEGG pathway #04012. Edges connecting nodes represent protein-protein interactions (PPI) from the Integrated Interactions Database (IID) that are supported by experimental evidence. Darker blue shading indicates targets that were more strongly suppressed in 3′UTR luciferase assays. (**E**) MesHMLE cells were stimulated with EGF and the activation of EGFR, AKT, MEK and ERK (or total protein) were assessed by western blotting in the presence or absence of miR-200c expression.

Given that EISA effectively differentiates between transcriptional and post-transcriptional regulation, we focused upon genes that were both strongly post-transcriptionally down-regulated in response to miR-200c and strongly predicted targets in order to identify the genes and processes that are directly regulated by miR-200c within the HMLE system. By examining the relationships between direct target genes using gene ontology, components of the EGFR signalling pathway were revealed to be the most strongly enriched, in addition to other ontologies that have well-established roles in EMT such as cell junction assembly (Figure 2A, Supplementary Data.xls). The majority of post-transcriptionally downregulated genes were predicted targets, with a higher overlap with predicted targets apparent for progressively more highly downregulated genes (Figure 2B). Closer examination revealed an abundance of targets whose gene products physically associate with the EGFR or that act within the AKT and ERK signalling pathways; many of which we validated using 3′UTR-luciferase reporter assays (Figure 2C, D). This suggests direct multi-faceted regulation of EGF signalling by miR-200c which is consistent with the epithelial-enforcing role of miR-200 given that EGF itself is a promoter of EMT (43–45) and that miR-200 had previously been implicated in the regulation of EGF signalling through the regulation of such genes as PLCG1 (46) and the EGFR itself (47). Accordingly, miR-200c strongly suppressed the activation of ERK (and MEK upstream of ERK) in response to EGF (Figure 2E). AKT was not activated by EGF within mesHMLE cells, precluding the examination of miR-200 as an AKT regulator, though in other cell lines (such as SHEP cells) where AKT is EGF-responsive, miR-200 dampened the AKT activation response as well. In SHEP cells, it is the ERK activation pathway that is largely uncoupled from canonical EGFR signalling (Supplementary Figure S1). Collectively, these data establish that miR-200c, at least in part, mediates biological effects through the direct, post-transcriptional and multi-component regulation of EGF-responsive signalling pathways.

The value of incorporating EISA into the study of miRNA-directed effects is shown by the increased significance of different seed-recognition elements among post-transcriptionally regulated genes (Figure 1D), and by the enrichment of pathways such as signalling downstream of the EGFR which are apparent after the incorporation of EISA into the analysis of miR-200c targets (Supplementary Data.xls), but that are otherwise masked by additional transcriptional effects in RNA-Seq.

### miR-200c co-ordinates a transcriptional response that underlies Mesenchymal-Epithelial Transition

**Figure 3.**
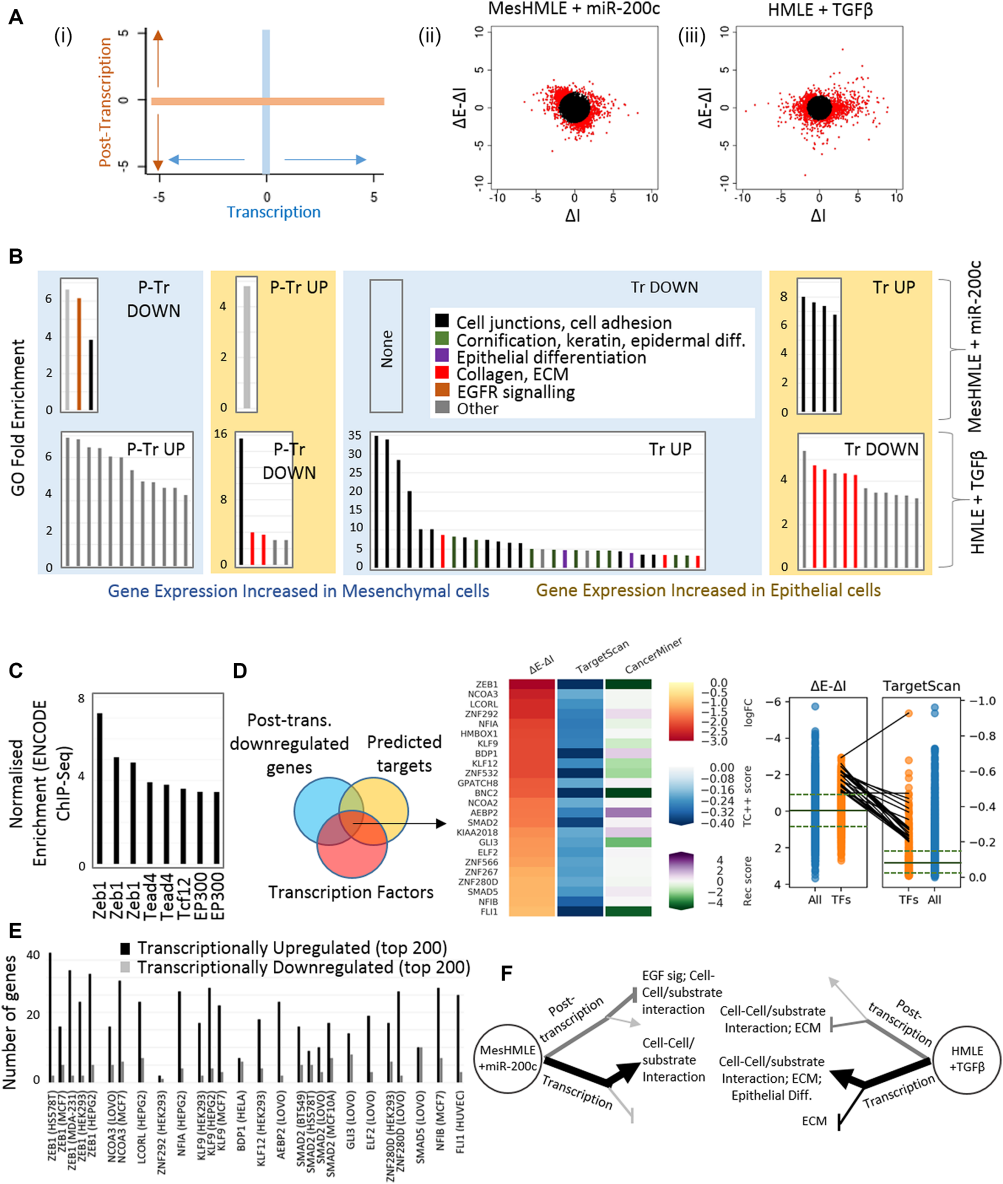
miR-200c and TGFβ co-ordinate a largely transcriptional response during EMT/MET (**A**). (i) ΔE – ΔI (post-transcription) was graphed against ΔI (transcription) to represent EISA-defined gene regulatory effects. Relative contributions of the two gene regulatory arms are shown after (ii) miR-200c expression in MesHMLE cells and (iii) TGFβ treatment of HMLEs. Red dots represent individual genes that were among the top 10% that were most regulated. The blacked out region represents the least changing 90% of genes. (**B**) Gene ontology (biological function) analyses were run on transcriptionally and post-transcriptionally up- and down-regulated genes in response to miR-200c and TGFβ. All ontologies with a fold enrichment over background >2 are shown. Colours represent functionally-related terms. To minimise ‘noisy’ enrichment of lowly-populated GO terms, as well as reducing the representation of very large and non-specific GO terms, all indicated GO terms possessed between 20 and 1500 genes and contained at least 5 GO-mapping genes within the gene list being interrogated. (**C**) Among transcriptionally upregulated genes, binding sites for mesenchymal-promoting transcription factors such as Zeb1 are enriched among ENCODE ChIP-Seq data (iRegulon) (33). Each bar represents an individual ChIP-Seq dataset. (**D**) TFs that are candidates to mediate downstream transcriptional changes are shown with their relative degree of post-transcriptional downregulation by miR-200c (ΔE – ΔI), the likelihood of their predicted targeting by miR-200c (TS) and the degree of their inversely correlated expression with miR-200c across breast cancer patients pooled from The Cancer Genome Atlas data (derived using CancerMiner (100)). Panel on right indicates the (ΔE - ΔI) and TS ranking of each of these TFs among all genes and all TFs. The solid line represents the median distribution relative to all genes. Dashed lines indicate upper and lower quartile distributions. (**E**) Number of genes among the top 200 most transcriptionally up- or down-regulated after miR-200 expression that are also identified within the top 1000 MACS-ranked peaks in human TF ChIP-Seq data (32). The cell line in which each experiment was performed is indicated. (**F**) Model for the co-ordinated regulatory processes underlying EMT/MET. Line depth and colour indicates relative extent of gene ontology enrichment.

Although the direct effects of miRNAs are exerted post-transcriptionally, such as the suppression of EGF signalling by miR-200c, many of the gene expression changes that occur during EMT/MET are in fact transcriptional, as indicated by the relative spread along the transcriptional (ΔI) and post-transcriptional (ΔE – ΔI) axes (Figure 3Ai) during miR-200c-driven MET (Figure 1C; Figure 3Aii). Transcriptional effects are even more pronounced during TGF-β–driven EMT (Figure 3Aiii). Although the interplay between miRNAs and transcription factors (TFs) are well established, a prevailing focus of miRNA research remains the understanding of function through the assessment of direct targets. EISA however, indicates that many of the responses to miR-200c are indirect (transcriptional). To probe the significance of these transcriptional changes, gene ontology (molecular function) analyses were performed separately for transcriptionally (and post-transcriptionally) up- and down-regulated gene sets in mesHMLE cells responding to miR-200c and in HMLE cells responding to TGFβ. Strikingly for miR-200c, the transcriptionally up-regulated genes contained the most enriched gene ontological terms (Figure 3b, Supplementary Data.xls). Furthermore, the function of these genes are of obvious relevance to EMT/MET (cell–cell junction assembly and organisation), indicating miR-200c-mediated transcriptional regulation is a key mechanism through which miR-200c promotes an epithelial phenotype, and suggesting more widely that the functionality of miRNAs may best be understood through broadening analysis beyond direct targets. This enrichment of ontological terms is unique to upregulated genes; there were no enriched ontologies among the genes that were transcriptionally decreased. This is consistent with the de-repression of genes that would otherwise be suppressed by mesenchymal-enforcing transcriptional repressors such as Zeb1, itself a prominent and direct miR-200c target (40–42).

The importance of ZEB1 as a mediator of miR-200c function is supported by the fact from among all of the ENCODE ChIP-Seq experiments, ZEB1 showed the greatest enrichment of protein binding in genes that were transcriptionally upregulated in response to miR-200c (Figure 3C). Although the miR-200:ZEB axis is a prominent regulator of EMT (39,41,48), ZEB1 alone is unlikely to fully account for the transcriptional response as almost three quarters of the genes that were transcriptionally upregulated in miR-200c expressing MesHMLE cells were not bound by ZEB1 within any of the three ENCODE ZEB1 ChIP-seq datasets (Supplementary Data.xls). Furthermore, the transcriptional controls of EMT are extensive (49) and less than 10% of all human transcriptional regulators are represented within ENCODE ChIP-Seq libraries. In order to suggest other candidate TFs that might co-mediate the miR-200c transcriptional response, we searched for TFs that were post-transcriptionally downregulated by miR-200c and that possess putative miR-200c binding sites within their 3′UTRs (top-ranking examples are shown in Figure 3D). TF target genes, derived from ChIP-Seq data for each of the TFs for which this was available (32), was then cross-referenced with the genes that were transcriptionally up- or down-regulated in response to miR-200 (Figure 3E). For the majority of TFs, which are mostly transcriptional repressors, TF targets are much more likely to be transcriptionally upregulated after miR-200 expression, consistent with the notion that multiple miR-200 regulated TFs suppress epithelial genes in a manner similar to Zeb. When comparing ontological enrichment between miR-200c expression (pro-MET) and TGF-β treatment (pro-EMT), it is noteworthy that there were more enriched ontologies for genes whose expressions are elevated in epithelial cells (upregulated by miR-200c or downregulated by TGF-β) and that the transcriptional arm of gene expression shows more evidence of co-ordinately regulating relevant functions than the post-transcriptional arm (Figure 3B). Nevertheless, both regulatory arms, and both miR-200c and TGF-β treatment, co-ordinate similar processes which have well established roles in the invasive aspects of EMT (cell-cell and cell-substrate interactions). In contrast, other aspects of EMT that are associated with collagen and the extracellular matrix (ECM), or with the epidermis (epidermal development, keratinisation and cornification), were uniquely associated with TGF-β (Figure 3B, F). Again, collagen/ECM and epidermal regulation were driven by both gene-regulatory arms, though their absence after miR-200 expression suggests the failure of this single miRNA to fully recapitulate all aspects of EMT that are driven in response to a physiological EMT stimulus.

As the exogenous expression of miRNAs can lead to non-physiological effects, we sought to further confirm the importance of indirect transcriptional regulation through inhibition of endogenous miR-200. In epithelial HMLE cells (Supplementary Figure S2), we observe that miR-200 inhibition exerts broad transcriptional effects. These in large part mirror miR-200 expression, with miR-200 inhibition de-repressing many of the same transcription factors that were downregulated by miR-200, in turn leading to the transcriptional repression of cell-junction associated genes. As inhibition of endogenous miRNAs typically have muted phenotypic effects, and given the HMLE cell model is of limited plasticity (likely due to epigenetic modification), we also inhibited the miR-200 family over a longer time course in a more malleable MDCK (canine) cell model (Supplementary Figure S3) (39,41,48). Again, we note a strong transcriptional response to miR-200 inhibition and find, as with TGF-β treatment of HMLE cells (Figure 3), genes associated with cell junctions, epidermal differentiation, cell migration, the actin cytoskeleton and TGF-β signalling are all transcriptionally regulated. Interestingly, although key transcription factors such as ZEB, BNC2 and GLI3 were miR-200 responsive in both HMLE and MDCK cells, many of the TFs upregulated by miR-200 inhibition in MDCKs differed from the HMLE system (Supplementary Figure S3). Further, the responsive TFs in both HMLE and MDCK cells also largely differed from the EMT-regulatory TFs reported in NMuMg (mouse) cells (49). Of the TFs identified as likely contributing to the transcriptional response to miR-200c in MesHMLE cells, the expression of one-third are more strongly associated with mesenchymal expression across TCGA (Supplementary Figure S4a). BNC2, a TF previously associated with a mesenchymal breast cancer stem cell phenotype (50), is particularly strongly mesenchymal specific (Supplementary Figure S4B–D), and is consistently responsive to miR-200c perturbation across cell lines (Figure 3, Supplementary Figures S2,S3). Collectively, these data suggest a model whereby key EMT regulators (like the ZEBs) play a consistent driving role across different systems, but also one where the nature of the supporting / accessory TFs are cell context dependent.

### Functionally significant transcriptional regulation is a widespread hallmark of miRNAs

**Figure 4.**
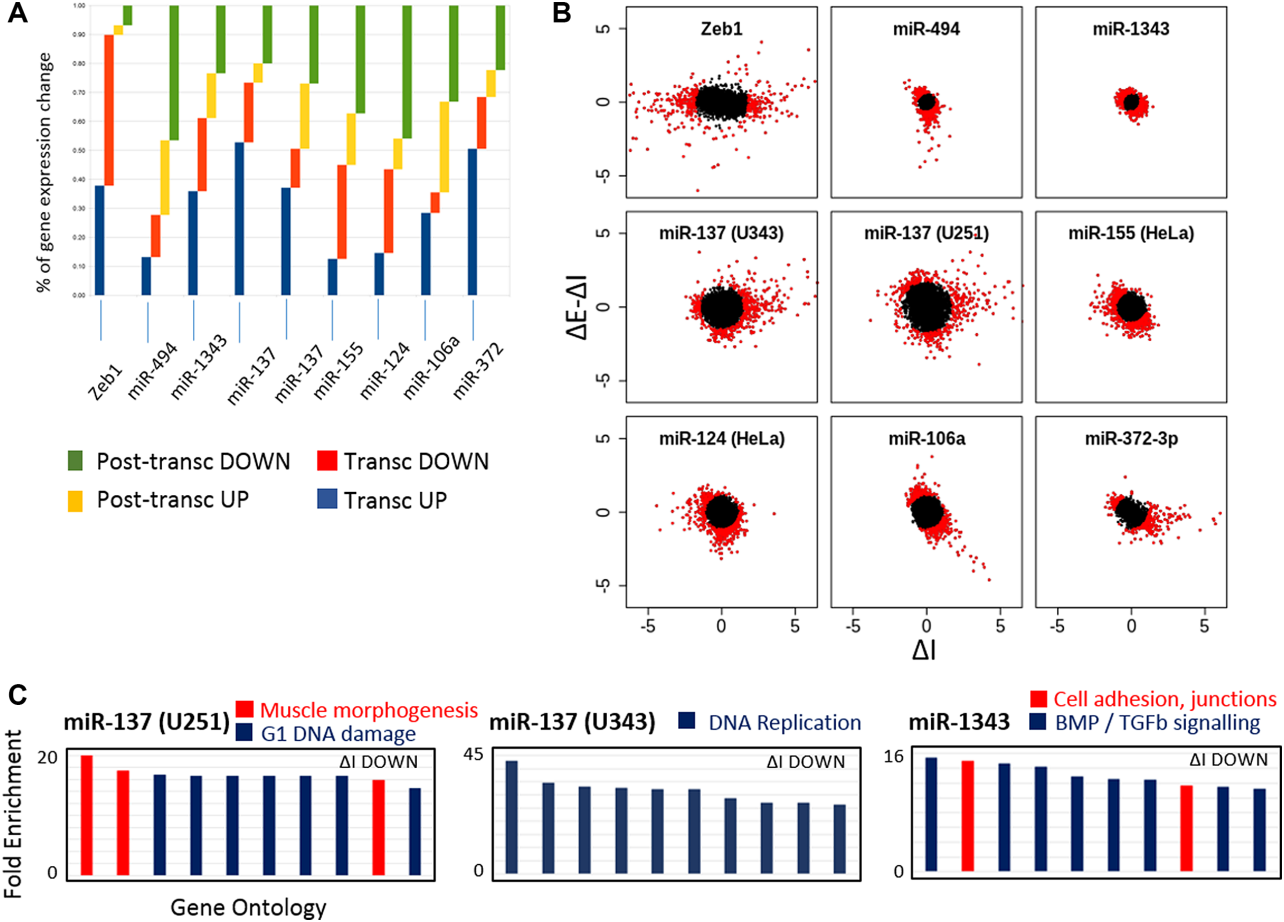
Co-ordinated, coherent transcriptional responses are widespread in response to many miRNAs. (**A**, **B**) The relative contributions of transcriptional and post-transcriptional mechanisms in response to the expression of ZEB1 or multiple individual miRNAs are shown by scatter plots (B) (ΔE – ΔI: ΔI) and quantitated in a bar histogram (A). The % gene expression change was calculated from among the top 10% of genes (red dots, B) that change in response to the miRNA in question. (**C**) Using the same GO-searching criteria described in Figure 3B, the 10 most enriched terms for each selected miRNA dataset are shown. Whether this is among transcriptionally up- or down-regulated genes are indicated.

After miR-200c perturbation, both the magnitude of the transcriptional response and the functional relationships of the transcriptionally responsive genes, suggest that indirect actions of miRNAs are centrally important to function. Whilst the transcriptional effects that result from miR-200c perturbation are likely mediated via multiple TFs (Figure 3D), it is well established that there is a particularly strong relationship between miR-200 and ZEB which might suggest that such a prominent transcriptional response is atypical of other miRNAs. Alternately, the fact that miRNA:TF feedback motifs are relatively common in genetic networks (8,10,13,51), and that the primary effect of Dicer knockout is transcriptional (52), suggests the transcriptional response to miRNAs may be an important, common and under-reported aspect of function. To investigate whether a prominent transcriptional response is common with miRNA perturbation, we subjected multiple datasets to EISA and found that of the 8 examined, between 27 and 73% of all gene expression changes were transcriptional (Figure 4A, B). For comparison, direct expression of the ZEB1 TF resulted in a 90% transcriptional response. Just as we had seen for miR-200c (Figure 3B), the nature of the transcriptionally regulated genes suggested that for many miRNAs, specific biological processes are co-ordinated within this transcriptional response (Figure 4C). Importantly, these processes that are being co-ordinated transcriptionally in response to the miRNA are often the same processes that the miRNA is known to regulate post-transcriptionally (see discussion for further details).

The implementation of EISA further allows examination of the broader mechanisms of gene regulation that occur in response to miRNA perturbation. By using EISA to separate the transcriptional and post-transcriptional response, we find that buffering between these two gene regulatory layers is extensive, with a tendency for transcriptionally upregulated genes to be post-transcriptionally suppressed (bottom right quadrant, Supplementary Figure S5), and conversely, for transcriptionally downregulated genes to be post-transcriptionally de-repressed (top left quadrant, Supplementary Figure S5). This is the case not only among direct miRNA targets, but across the entire transcriptomic response to miRNA perturbation, which collectively reinforces our conclusions that whilst the direct and strong post-transcriptional targeting of key genes is a prominent feature of miRNA function, and is the focus of most miRNA literature, these events happen alongside co-ordinated transcriptional modulation and within a broader context of extensive homeostatic buffering. We also note for several miRNAs (such as miR-200c), a subset of genes are clearly inhibited through both gene regulatory layers (Supplementary Figure S5, bottom left quadrants), indicating co-ordinated actions where indirect transcriptional responses reinforce direct post-transcriptional gene suppression. Collectively, these observations suggest that indirect transcriptional effects are fundamental to the roles that miRNAs play and demonstrate the importance of taking into account these indirect effects.

## DISCUSSION

In this study, the application of EISA reveals the importance of assessing the indirect effects of miRNAs on transcription. The overarching concept of a transcriptional response to miRNAs is expected as there are numerous reports of miRNA: TF co-regulatory relationships, and global disruption of miRNA biogenesis (Dicer knockout) results in a majority transcriptional response (52). What is unexpected however, is the magnitude of the transcriptional response that occurs even after the perturbation of single miRNAs, and that the nature of genes that are co-ordinated at the transcriptional level appear to explain many aspects of the function of the miRNA itself. This is demonstrated with regard to miR-200c and the enforcement of an epithelial phenotype (Figure 3, Supplementary Figures S2, S3) and more widely implied for other miRNAs (Figure 4). For example, genes associated with cell cycle control at the G1 checkpoint were prominently over-represented among transcriptionally down-regulated genes in response to miR-137. The induction of G1 arrest by miR-137 has been widely reported (53–58), though in each case (as in typical in miRNA studies), the mechanistic explanation for the observation has been attributed to individual genes (AEG1 (54), Cdc42 (55), SRC3 (58), ESRRA (57)) directly targeted by miR-137 post-transcriptionally. The same is true of the transcriptional downregulation of genes associated with BMP/TGF-β signalling in response to miR-1343 (59,60). Interestingly, of only three publications involving miR-1343, two detail an effect on TGF-β signaling, with the only mechanistic explanations to date involving direct, post-transcriptional targeting of SMAD2 and SMAD3 (60). Together, these results suggest that often the indirect transcriptional effects comprise an unexpectedly large fraction of the coordinated functional impact of miRNAs.

The repertoire of gene regulatory mechanisms is extensive and there are now various reports characterising transcriptional and post-transcriptional regulation of the same gene (61), or transcriptional and post-transcriptional regulation of downstream processes initiated by a single protein (62–64). On a broader scale, the co-ordination of transcription and co-transcriptional processes such as alternate splicing, 5′-capping and 3′-end formation are also established as they are both spatially and temporally coupled (65–69). Although our observations are conceptually similar, the functional linkage of transcription and post-transcriptional mRNA stability that we find, despite occurring in separate cellular compartments, suggests a level of ‘cross-talk’ between gene regulatory arms which is only now starting to become appreciated in light of the methodological improvements required to perform such analyses. Examples of new insights afforded by such advancements is a recent report showing that during circadian oscillations, only a subset of genes that are rhythmically expressed are themselves rhythmically transcribed, suggesting an important post-transcriptional contribution (70,71). Similarly, co-ordinated transcriptional and mRNA stability effects occur within the Ataxia Telangiectasia Mutated (ATM)- and p53-regulated ionising radiation response (72).

Our observations here indicate that a strong transcriptional component is seen across all miRNA perturbation datasets examined (Figure 4), including where endogenous miRNAs have been inhibited, and that this transcriptional response is both a prominent driver of phenotypic outcomes, and a major component of a broad transcriptomic buffering response that is pervasive throughout the transcriptome in response to miRNA expression. (Supplementary Figure S5). Widespread transcriptional buffering however is not universal, as demonstrated by miR-137 expression (Supplementary Figure S5). Whilst the reasons for this are not definitive, it is noteworthy that among the miRNAs examined, miR-137 is not encoded within a polycistronic cluster. Hence, the expression of miR-137 is less likely to lead to prominent effects on the expression of other clustered miRNAs with which it is typically co-expressed, as occurs with the upregulation of miR-200c for example, where the suppression of ZEB would de-repress not only endogenous miR-200c, but also the four other clustered miR-200 family members (39). Gene expression effects (we speculate) may therefore not be as easily potentiated across the post-transcriptional regulatory landscape by the manipulation of isolated miRNAs such as miR-137.

These conclusions are made possible through the implementation of EISA, though other methodologies are available to specifically examine the rate of transcription including Nascent-Seq (68,69,73), whereby newly transcribed RNAs are co-purified with transcription complexes, global-run on sequencing (GRO-Seq) (74), in which nascent transcripts are identified through their incorporation of nucleotide analogs, and nuclear fractionation to enrich for new transcripts prior to their export into the cytoplasm (75). A great benefit of EISA is that it allows this information to be extracted from existing sequencing datasets, provided they are of sufficient depth, and does not require implementation of difficult and specialist protocols that themselves may introduce additional biases. Further, the additional benefit that other methods do not natively provide is the simultaneous inference of post-transcriptional regulation levels, which is particularly useful for miRNA research where identification of putative direct targets is often left to computational prediction alone.

Critical to the success of EISA is the assumption that the measurement of intronic sequencing reads offers an accurate measure of nascent transcription. Several lines of evidence support this: Firstly, there is no evidence of independent intronic transcriptional units using methods that specifically profile nascent transcription (76). Secondly, intronic and exonic reads were correlated for genes that are transcriptionally targeted, yet as expected, only post-transcriptional regulation was observed for genes targeted by transfected siRNAs (22). Thirdly, EISA-associated intronic reads are highly associated with histone modifications (Figure 1, (22,52)) that themselves are effective markers of transcriptional activity (77,78). This provides strong experimental support for the interpretation of EISA on a global scale, though it remains true that at the single gene level, alternate splicing or non-coding RNAs could cause misinterpretation of the intronic signal without further investigation.

Since publication of the methodology (22), EISA has been utilised to probe the gene regulatory mechanisms in response to the knock-down of specific genes (79–82), drugs or other extracellular stimuli (83–85), circadian rhythms (86) and developmental processes (87), or to assist with the identification of direct miRNA targets from complex sequencing datasets (37,52). This work provides a further demonstration of EISA’s capacity to offer insight into gene regulatory mechanisms, being used in this case to reveal the co-ordinated, complementary activities of the transcriptional and post-transcriptional gene regulatory arms in Epithelial–Mesenchymal and Mesenchymal–Epithelial Transitions.

It is noteworthy that although EGFR signalling pathway genes were strongly post-transcriptionally downregulated in response to miR-200c (Figures 2A, 3B), a parallel post-transcriptional upregulation was not noted among the ontologies that were enriched within TGF-β driven EMT (Figure 3B). Upon closer examination, we see that almost all of the miR-200c responsive genes within this pathway were post-transcriptionally up-regulated after TGF-β treatment, however most did not rise to a level of upregulation sufficient to place them in the top 5% of TGF-β regulated genes (the cut-off used in Figure 3B). Hence, this signalling pathway was not identified as being enriched in the TGF-β response. One possible explanation is that although signalling downstream of EGFR is one of the major pathways regulated by miR-200c, miR-200c is but one of multiple miRNAs that are regulated in response to TGF-β, and this response is swamped amongst the plethora of other gene regulatory events that occur, at least at the timepoint at which the experiment was conducted. The capacity to look in granular detail at such gene regulatory responses showcases the insight made possible by using EISA and allows us to conclude that whilst TGF-β driven EMT and miR-200c driven MET have clear parallels, they are not merely identical and opposite switches.

To be clear, our findings do not imply that miRNAs are not important post-transcriptional regulators. Indeed, we use EISA to identify extensive, direct post-transcriptional targeting of the EGFR-signalling pathway by miR-200c (Figure 2), noting particularly strong suppression of EGFR-regulators including DCBLD2 (88), ERRFI1 (89) and PTPN12 (90). Instead, we argue that in addition to direct post-transcriptional regulation, miRNAs exert many of their roles indirectly, via key targets (TFs) that themselves exert network-wide regulatory effects, co-ordinating transcriptional responses of groups of functionally related genes that serve as effectors of miRNA function. MiRNA: TF motifs, which are over-represented within genetic networks (9,10,91–93), are ideally suited to elicit rapid and profound changes to gene expression as transcripts encoding TFs are generally lowly expressed, TF mRNAs and proteins often have short half-lives and the transcriptional response to changing levels of TFs is rapid (94–99). The conclusions we draw further demonstrate the importance of considering ‘indirect’ effects, which are central to miRNA function but can be ignored if direct targets are the sole focus. Our study highlights the power of EISA for analysing both levels of regulation, separately yet simultaneously.

## Supplementary Material

gkz664_Supplemental_Files

## References

[1] Osella M., Bosia C., Cora D., Caselle M. The role of incoherent microRNA-mediated feedforward loops in noise buffering. PLoS Comput. Biol. 2011; 7:e1001101. 21423718 10.1371/journal.pcbi.1001101 PMC3053320

[2] Riba A., Bosia C., El Baroudi M., Ollino L., Caselle M. A combination of transcriptional and microRNA regulation improves the stability of the relative concentrations of target genes. PLoS Comput. Biol. 2014; 10:e1003490. 24586138 10.1371/journal.pcbi.1003490 PMC3937125

[3] Siciliano V., Garzilli I., Fracassi C., Criscuolo S., Ventre S., di Bernardo D. MiRNAs confer phenotypic robustness to gene networks by suppressing biological noise. Nat. Commun. 2013; 4:2364. 24077216 10.1038/ncomms3364 PMC3836244

[4] Zhang H., Chen Y., Chen Y. Noise propagation in gene regulation networks involving interlinked positive and negative feedback loops. PLoS One. 2012; 7:e51840. 23284787 10.1371/journal.pone.0051840 PMC3527455

[5] Mukherji S., Ebert M.S., Zheng G.X., Tsang J.S., Sharp P.A., van Oudenaarden A. MicroRNAs can generate thresholds in target gene expression. Nat. Genet. 2011; 43:854–859. 21857679 10.1038/ng.905 PMC3163764

[6] Arora S., Rana R., Chhabra A., Jaiswal A., Rani V. miRNA-transcription factor interactions: a combinatorial regulation of gene expression. Mol. Genet. Genomics. 2013; 288:77–87. 23334784 10.1007/s00438-013-0734-z

[7] Hobert O. Gene regulation by transcription factors and microRNAs. Science. 2008; 319:1785–1786. 18369135 10.1126/science.1151651

[8] Re A., Cora D., Taverna D., Caselle M. Genome-wide survey of microRNA-transcription factor feed-forward regulatory circuits in human. Mol. Biosyst. 2009; 5:854–867. 19603121 10.1039/b900177h PMC2898627

[9] Shalgi R., Lieber D., Oren M., Pilpel Y. Global and local architecture of the mammalian microRNA-transcription factor regulatory network. PLoS Comput. Biol. 2007; 3:e131. 17630826 10.1371/journal.pcbi.0030131 PMC1914371

[10] Tsang J., Zhu J., van Oudenaarden A. MicroRNA-mediated feedback and feedforward loops are recurrent network motifs in mammals. Mol. Cell. 2007; 26:753–767. 17560377 10.1016/j.molcel.2007.05.018 PMC2072999

[11] Zhou Y., Ferguson J., Chang J.T., Kluger Y. Inter- and intra-combinatorial regulation by transcription factors and microRNAs. BMC Genomics. 2007; 8:396. 17971223 10.1186/1471-2164-8-396 PMC2206040

[12] Bisognin A., Sales G., Coppe A., Bortoluzzi S., Romualdi C. MAGIA(2): from miRNA and genes expression data integrative analysis to microRNA-transcription factor mixed regulatory circuits (2012 update). Nucleic Acids Res. 2012; 40:W13–W21. 22618880 10.1093/nar/gks460 PMC3394337

[13] Friard O., Re A., Taverna D., De Bortoli M., Cora D. CircuitsDB: a database of mixed microRNA/transcription factor feed-forward regulatory circuits in human and mouse. BMC Bioinformatics. 2010; 11:435. 20731828 10.1186/1471-2105-11-435 PMC2936401

[14] Liu Z.P., Wu C., Miao H., Wu H. RegNetwork: an integrated database of transcriptional and post-transcriptional regulatory networks in human and mouse. Database. 2015; 2015:bav095. 26424082 10.1093/database/bav095 PMC4589691

[15] Vlachos I.S., Vergoulis T., Paraskevopoulou M.D., Lykokanellos F., Georgakilas G., Georgiou P., Chatzopoulos S., Karagkouni D., Christodoulou F., Dalamagas T. et al. DIANA-mirExTra v2.0: Uncovering microRNAs and transcription factors with crucial roles in NGS expression data. Nucleic Acids Res. 2016; 44:W128–W134. 27207881 10.1093/nar/gkw455 PMC4987956

[16] Wang J., Lu M., Qiu C., Cui Q. TransmiR: a transcription factor-microRNA regulation database. Nucleic Acids Res. 2010; 38:D119–D122. 19786497 10.1093/nar/gkp803 PMC2808874

[17] Zhang H.M., Kuang S., Xiong X., Gao T., Liu C., Guo A.Y. Transcription factor and microRNA co-regulatory loops: important regulatory motifs in biological processes and diseases. Brief. Bioinform. 2015; 16:45–58. 24307685 10.1093/bib/bbt085

[18] Voon D.C., Huang R.Y., Jackson R.A., Thiery J.P. The EMT spectrum and therapeutic opportunities. Mol. Oncol. 2017; 11:878–891. 28544151 10.1002/1878-0261.12082 PMC5496500

[19] Jolly M.K., Boareto M., Huang B., Jia D., Lu M., Ben-Jacob E., Onuchic J.N., Levine H. Implications of the hybrid epithelial/mesenchymal phenotype in metastasis. Front. Oncol. 2015; 5:155. 26258068 10.3389/fonc.2015.00155 PMC4507461

[20] Lu M., Jolly M.K., Levine H., Onuchic J.N., Ben-Jacob E. MicroRNA-based regulation of epithelial-hybrid-mesenchymal fate determination. Proc. Natl. Acad. Sci. U.S.A. 2013; 110:18144–18149. 24154725 10.1073/pnas.1318192110 PMC3831488

[21] Tian X.J., Zhang H., Xing J. Coupled reversible and irreversible bistable switches underlying TGFbeta-induced epithelial to mesenchymal transition. Biophys. J. 2013; 105:1079–1089. 23972859 10.1016/j.bpj.2013.07.011 PMC3752104

[22] Gaidatzis D., Burger L., Florescu M., Stadler M.B. Analysis of intronic and exonic reads in RNA-seq data characterizes transcriptional and post-transcriptional regulation. Nat. Biotechnol. 2015; 33:722–729. 26098447 10.1038/nbt.3269

[23] Mani S.A., Guo W., Liao M.J., Eaton E.N., Ayyanan A., Zhou A.Y., Brooks M., Reinhard F., Zhang C.C., Shipitsin M. et al. The epithelial-mesenchymal transition generates cells with properties of stem cells. Cell. 2008; 133:704–715. 18485877 10.1016/j.cell.2008.03.027 PMC2728032

[24] Martin M. Cutadapt removes adapter sequences from high-throughput sequencing reads. EMBnet.journal. 2011; 17:doi:10.14806/ej.17.1.200.

[25] Dobin A., Davis C.A., Schlesinger F., Drenkow J., Zaleski C., Jha S., Batut P., Chaisson M., Gingeras T.R. STAR: ultrafast universal RNA-seq aligner. Bioinformatics. 2013; 29:15–21. 23104886 10.1093/bioinformatics/bts635 PMC3530905

[26] Thorvaldsdottir H., Robinson J.T., Mesirov J.P. Integrative Genomics Viewer (IGV): high-performance genomics data visualization and exploration. Brief. Bioinform. 2013; 14:178–192. 22517427 10.1093/bib/bbs017 PMC3603213

[27] Cursons J., Pillman K.A., Scheer K.G., Gregory P.A., Foroutan M., Hediyeh-Zadeh S., Toubia J., Crampin E.J., Goodall G.J., Bracken C.P. et al. Combinatorial targeting by MicroRNAs co-ordinates post-transcriptional control of EMT. Cell Syst. 2018; 7:77–91. 30007539 10.1016/j.cels.2018.05.019

[28] Robinson M.D., McCarthy D.J., Smyth G.K. edgeR: a Bioconductor package for differential expression analysis of digital gene expression data. Bioinformatics. 2010; 26:139–140. 19910308 10.1093/bioinformatics/btp616 PMC2796818

[29] Ritchie M.E., Phipson B., Wu D., Hu Y., Law C.W., Shi W., Smyth G.K. limma powers differential expression analyses for RNA-sequencing and microarray studies. Nucleic Acids Res. 2015; 43:e47. 25605792 10.1093/nar/gkv007 PMC4402510

[30] Conn S.J., Pillman K.A., Toubia J., Conn V.M., Salmanidis M., Phillips C.A., Roslan S., Schreiber A.W., Gregory P.A., Goodall G.J. The RNA binding protein quaking regulates formation of circRNAs. Cell. 2015; 160:1125–1134. 25768908 10.1016/j.cell.2015.02.014

[31] Pillman K.A., Phillips C.A., Roslan S., Toubia J., Dredge B.K., Bert A.G., Lumb R., Neumann D.P., Li X., Conn S.J. et al. miR-200/375 control epithelial plasticity-associated alternative splicing by repressing the RNA-binding protein quaking. EMBO J. 2018; 37:e99016. 29871889 10.15252/embj.201899016 PMC6028027

[32] Yevshin I., Sharipov R., Kolmykov S., Kondrakhin Y., Kolpakov F. GTRD: a database on gene transcription regulation-2019 update. Nucleic Acids Res. 2019; 47:D100–D105. 30445619 10.1093/nar/gky1128 PMC6323985

[33] Janky R., Verfaillie A., Imrichova H., Van de Sande B., Standaert L., Christiaens V., Hulselmans G., Herten K., Naval Sanchez M., Potier D. et al. iRegulon: from a gene list to a gene regulatory network using large motif and track collections. PLoS Comput. Biol. 2014; 10:e1003731. 25058159 10.1371/journal.pcbi.1003731 PMC4109854

[34] Consortium E.P. A user's guide to the encyclopedia of DNA elements (ENCODE). PLoS Biol. 2011; 9:e1001046. 21526222 10.1371/journal.pbio.1001046 PMC3079585

[35] Zhang Y., Liu T., Meyer C.A., Eeckhoute J., Johnson D.S., Bernstein B.E., Nusbaum C., Myers R.M., Brown M., Li W. et al. Model-based analysis of ChIP-Seq (MACS). Genome Biol. 2008; 9:R137. 18798982 10.1186/gb-2008-9-9-r137 PMC2592715

[36] Shao Z., Zhang Y., Yuan G.C., Orkin S.H., Waxman D.J. MAnorm: a robust model for quantitative comparison of ChIP-Seq data sets. Genome Biol. 2012; 13:R16. 22424423 10.1186/gb-2012-13-3-r16 PMC3439967

[37] Hilz S., Fogarty E.A., Modzelewski A.J., Cohen P.E., Grimson A. Transcriptome profiling of the developing male germ line identifies the miR-29 family as a global regulator during meiosis. RNA Biol. 2017; 14:219–235. 27981880 10.1080/15476286.2016.1270002 PMC5324742

[38] Brabletz S., Brabletz T. The ZEB/miR-200 feedback loop–a motor of cellular plasticity in development and cancer. EMBO Rep. 2010; 11:670–677. 20706219 10.1038/embor.2010.117 PMC2933868

[39] Bracken C.P., Gregory P.A., Kolesnikoff N., Bert A.G., Wang J., Shannon M.F., Goodall G.J. A double-negative feedback loop between ZEB1-SIP1 and the microRNA-200 family regulates epithelial-mesenchymal transition. Cancer Res. 2008; 68:7846–7854. 18829540 10.1158/0008-5472.CAN-08-1942

[40] Burk U., Schubert J., Wellner U., Schmalhofer O., Vincan E., Spaderna S., Brabletz T. A reciprocal repression between ZEB1 and members of the miR-200 family promotes EMT and invasion in cancer cells. EMBO Rep. 2008; 9:582–589. 18483486 10.1038/embor.2008.74 PMC2396950

[41] Gregory P.A., Bert A.G., Paterson E.L., Barry S.C., Tsykin A., Farshid G., Vadas M.A., Khew-Goodall Y., Goodall G.J. The miR-200 family and miR-205 regulate epithelial to mesenchymal transition by targeting ZEB1 and SIP1. Nat. Cell Biol. 2008; 10:593–601. 18376396 10.1038/ncb1722

[42] Park S.M., Gaur A.B., Lengyel E., Peter M.E. The miR-200 family determines the epithelial phenotype of cancer cells by targeting the E-cadherin repressors ZEB1 and ZEB2. Genes Dev. 2008; 22:894–907. 18381893 10.1101/gad.1640608 PMC2279201

[43] Grande M., Franzen A., Karlsson J.O., Ericson L.E., Heldin N.E., Nilsson M. Transforming growth factor-beta and epidermal growth factor synergistically stimulate epithelial to mesenchymal transition (EMT) through a MEK-dependent mechanism in primary cultured pig thyrocytes. J. Cell Sci. 2002; 115:4227–4236. 12376555 10.1242/jcs.00091

[44] Lu Z., Ghosh S., Wang Z., Hunter T. Downregulation of caveolin-1 function by EGF leads to the loss of E-cadherin, increased transcriptional activity of beta-catenin, and enhanced tumor cell invasion. Cancer Cell. 2003; 4:499–515. 14706341 10.1016/s1535-6108(03)00304-0

[45] Pagan R., Martin I., Llobera M., Vilaro S. Epithelial-mesenchymal transition of cultured rat neonatal hepatocytes is differentially regulated in response to epidermal growth factor and dimethyl sulfoxide. Hepatology. 1997; 25:598–606. 9049205 10.1002/hep.510250318

[46] Uhlmann S., Zhang J.D., Schwager A., Mannsperger H., Riazalhosseini Y., Burmester S., Ward A., Korf U., Wiemann S., Sahin O. miR-200bc/429 cluster targets PLCgamma1 and differentially regulates proliferation and EGF-driven invasion than miR-200a/141 in breast cancer. Oncogene. 2010; 29:4297–4306. 20514023 10.1038/onc.2010.201

[47] Bracken C.P., Li X., Wright J.A., Lawrence D.M., Pillman K.A., Salmanidis M., Anderson M.A., Dredge B.K., Gregory P.A., Tsykin A. et al. Genome-wide identification of miR-200 targets reveals a regulatory network controlling cell invasion. EMBO J. 2014; 33:2040–2056. 25069772 10.15252/embj.201488641 PMC4195771

[48] Gregory P.A., Bracken C.P., Smith E., Bert A.G., Wright J.A., Roslan S., Morris M., Wyatt L., Farshid G., Lim Y.Y. et al. An autocrine TGF-beta/ZEB/miR-200 signaling network regulates establishment and maintenance of epithelial-mesenchymal transition. Mol. Biol. Cell. 2011; 22:1686–1698. 21411626 10.1091/mbc.E11-02-0103 PMC3093321

[49] Meyer-Schaller N., Cardner M., Diepenbruck M., Saxena M., Tiede S., Luond F., Ivanek R., Beerenwinkel N., Christofori G. A hierarchical regulatory landscape during the multiple stages of EMT. Dev. Cell. 2019; 48:539–553. 30713070 10.1016/j.devcel.2018.12.023

[50] da Silveira W.A., Palma P.V.B., Sicchieri R.D., Villacis R.A.R., Mandarano L.R.M., Oliveira T.M.G., Antonio H.M.R., Andrade J.M., Muglia V.F., Rogatto S.R. et al. Transcription factor networks derived from breast cancer stem cells control the immune response in the Basal subtype. Sci. Rep. 2017; 7:2851. 28588211 10.1038/s41598-017-02761-6 PMC5460106

[51] Martinez N.J., Ow M.C., Barrasa M.I., Hammell M., Sequerra R., Doucette-Stamm L., Roth F.P., Ambros V.R., Walhout A.J. A C. elegans genome-scale microRNA network contains composite feedback motifs with high flux capacity. Genes Dev. 2008; 22:2535–2549. 18794350 10.1101/gad.1678608 PMC2546694

[52] Gosline S.J., Gurtan A.M., JnBaptiste C.K., Bosson A., Milani P., Dalin S., Matthews B.J., Yap Y.S., Sharp P.A., Fraenkel E. Elucidating MicroRNA regulatory networks using transcriptional, post-transcriptional, and histone modification measurements. Cell Rep. 2016; 14:310–319. 26748710 10.1016/j.celrep.2015.12.031 PMC4831719

[53] Chen R., Zhang Y., Zhang C., Wu H., Yang S. miR-137 inhibits the proliferation of human non-small cell lung cancer cells by targeting SRC3. Oncol Lett. 2017; 13:3905–3911. 28521488 10.3892/ol.2017.5904 PMC5431322

[54] Guo J., Xia B., Meng F., Lou G. miR-137 suppresses cell growth in ovarian cancer by targeting AEG-1. Biochem. Biophys. Res. Commun. 2013; 441:357–363. 24144591 10.1016/j.bbrc.2013.10.052

[55] Liu M., Lang N., Qiu M., Xu F., Li Q., Tang Q., Chen J., Chen X., Zhang S., Liu Z. et al. miR-137 targets Cdc42 expression, induces cell cycle G1 arrest and inhibits invasion in colorectal cancer cells. Int. J. Cancer. 2011; 128:1269–1279. 20473940 10.1002/ijc.25452

[56] Zhang L., Li Z., Gai F., Wang Y. MicroRNA-137 suppresses tumor growth in epithelial ovarian cancer in vitro and in vivo. Mol. Med. Rep. 2015; 12:3107–3114. 25955305 10.3892/mmr.2015.3756

[57] Zhao Y., Li Y., Lou G., Zhao L., Xu Z., Zhang Y., He F. MiR-137 targets estrogen-related receptor alpha and impairs the proliferative and migratory capacity of breast cancer cells. PLoS One. 2012; 7:e39102. 22723937 10.1371/journal.pone.0039102 PMC3377602

[58] Zhu X., Li Y., Shen H., Li H., Long L., Hui L., Xu W. miR-137 inhibits the proliferation of lung cancer cells by targeting Cdc42 and Cdk6. FEBS Lett. 2013; 587:73–81. 23178712 10.1016/j.febslet.2012.11.004

[59] Stolzenburg L.R., Harris A. Microvesicle-mediated delivery of miR-1343: impact on markers of fibrosis. Cell Tissue Res. 2018; 371:325–338. 29022142 10.1007/s00441-017-2697-6 PMC5809304

[60] Stolzenburg L.R., Wachtel S., Dang H., Harris A. miR-1343 attenuates pathways of fibrosis by targeting the TGF-beta receptors. Biochem. J. 2016; 473:245–256. 26542979 10.1042/BJ20150821 PMC4867233

[61] Zhao C., Isenberg J.S., Popel A.S. Transcriptional and post-transcriptional regulation of thrombospondin-1 expression: a computational model. PLoS Comput. Biol. 2017; 13:e1005272. 28045898 10.1371/journal.pcbi.1005272 PMC5207393

[62] Hu Y.J., Belaghzal H., Hsiao W.Y., Qi J., Bradner J.E., Guertin D.A., Sif S., Imbalzano A.N. Transcriptional and post-transcriptional control of adipocyte differentiation by Jumonji domain-containing protein 6. Nucleic Acids Res. 2015; 43:7790–7804. 26117538 10.1093/nar/gkv645 PMC4652747

[63] Koh C.M., Iwata T., Zheng Q., Bethel C., Yegnasubramanian S., De Marzo A.M. Myc enforces overexpression of EZH2 in early prostatic neoplasia via transcriptional and post-transcriptional mechanisms. Oncotarget. 2011; 2:669–683. 21941025 10.18632/oncotarget.327 PMC3248223

[64] Park P.H., Huang H., McMullen M.R., Mandal P., Sun L., Nagy L.E. Suppression of lipopolysaccharide-stimulated tumor necrosis factor-alpha production by adiponectin is mediated by transcriptional and post-transcriptional mechanisms. J. Biol. Chem. 2008; 283:26850–26858. 18678874 10.1074/jbc.M802787200 PMC2556004

[65] Bentley D.L. Coupling mRNA processing with transcription in time and space. Nat. Rev. Genet. 2014; 15:163–175. 24514444 10.1038/nrg3662 PMC4304646

[66] Li W., Park J.Y., Zheng D., Hoque M., Yehia G., Tian B. Alternative cleavage and polyadenylation in spermatogenesis connects chromatin regulation with post-transcriptional control. BMC Biol. 2016; 14:6. 26801249 10.1186/s12915-016-0229-6 PMC4724118

[67] Nojima T., Gomes T., Grosso A.R.F., Kimura H., Dye M.J., Dhir S., Carmo-Fonseca M., Proudfoot N.J. Mammalian NET-Seq reveals genome-wide nascent transcription coupled to RNA processing. Cell. 2015; 161:526–540. 25910207 10.1016/j.cell.2015.03.027 PMC4410947

[68] Khodor Y.L., Rodriguez J., Abruzzi K.C., Tang C.H., Marr M.T. 2nd, Rosbash M. Nascent-seq indicates widespread cotranscriptional pre-mRNA splicing in Drosophila. Genes Dev. 2011; 25:2502–2512. 22156210 10.1101/gad.178962.111 PMC3243060

[69] Rodriguez J., Menet J.S., Rosbash M. Nascent-seq indicates widespread cotranscriptional RNA editing in Drosophila. Mol. Cell. 2012; 47:27–37. 22658416 10.1016/j.molcel.2012.05.002 PMC3409466

[70] Menet J.S., Rodriguez J., Abruzzi K.C., Rosbash M. Nascent-Seq reveals novel features of mouse circadian transcriptional regulation. Elife. 2012; 1:e00011. 23150795 10.7554/eLife.00011 PMC3492862

[71] Rodriguez J., Tang C.H., Khodor Y.L., Vodala S., Menet J.S., Rosbash M. Nascent-Seq analysis of Drosophila cycling gene expression. Proc. Natl. Acad. Sci. U.S.A. 2013; 110:E275–284. 23297234 10.1073/pnas.1219969110 PMC3557077

[72] Venkata Narayanan I., Paulsen M.T., Bedi K., Berg N., Ljungman E.A., Francia S., Veloso A., Magnuson B., di Fagagna F.D., Wilson T.E. et al. Transcriptional and post-transcriptional regulation of the ionizing radiation response by ATM and p53. Sci. Rep. 2017; 7:43598. 28256581 10.1038/srep43598 PMC5335570

[73] Core L.J., Waterfall J.J., Lis J.T. Nascent RNA sequencing reveals widespread pausing and divergent initiation at human promoters. Science. 2008; 322:1845–1848. 19056941 10.1126/science.1162228 PMC2833333

[74] Danko C.G., Hyland S.L., Core L.J., Martins A.L., Waters C.T., Lee H.W., Cheung V.G., Kraus W.L., Lis J.T., Siepel A. Identification of active transcriptional regulatory elements from GRO-seq data. Nat. Methods. 2015; 12:433–438. 25799441 10.1038/nmeth.3329 PMC4507281

[75] Zaghlool A., Ameur A., Nyberg L., Halvardson J., Grabherr M., Cavelier L., Feuk L. Efficient cellular fractionation improves RNA sequencing analysis of mature and nascent transcripts from human tissues. BMC Biotechnol. 2013; 13:99. 24225116 10.1186/1472-6750-13-99 PMC3833653

[76] Ameur A., Zaghlool A., Halvardson J., Wetterbom A., Gyllensten U., Cavelier L., Feuk L. Total RNA sequencing reveals nascent transcription and widespread co-transcriptional splicing in the human brain. Nat. Struct. Mol. Biol. 2011; 18:1435–1440. 22056773 10.1038/nsmb.2143

[77] Karlic R., Chung H.R., Lasserre J., Vlahovicek K., Vingron M. Histone modification levels are predictive for gene expression. Proc. Natl. Acad. Sci. U.S.A. 2010; 107:2926–2931. 20133639 10.1073/pnas.0909344107 PMC2814872

[78] Tippmann S.C., Ivanek R., Gaidatzis D., Scholer A., Hoerner L., van Nimwegen E., Stadler P.F., Stadler M.B., Schubeler D. Chromatin measurements reveal contributions of synthesis and decay to steady-state mRNA levels. Mol. Syst. Biol. 2012; 8:593. 22806141 10.1038/msb.2012.23 PMC3421439

[79] Akay A., Di Domenico T., Suen K.M., Nabih A., Parada G.E., Larance M., Medhi R., Berkyurek A.C., Zhang X., Wedeles C.J. et al. The helicase Aquarius/EMB-4 is required to overcome intronic barriers to allow nuclear RNAi pathways to heritably silence transcription. Dev. Cell. 2017; 42:241–255. 28787591 10.1016/j.devcel.2017.07.002 PMC5554785

[80] Habacher C., Guo Y., Venz R., Kumari P., Neagu A., Gaidatzis D., Harvald E.B., Faergeman N.J., Gut H., Ciosk R. Ribonuclease-Mediated control of body fat. Dev. Cell. 2016; 39:359–369. 27746047 10.1016/j.devcel.2016.09.018

[81] Iasillo C., Schmid M., Yahia Y., Maqbool M.A., Descostes N., Karadoulama E., Bertrand E., Andrau J.C., Jensen T.H. ARS2 is a general suppressor of pervasive transcription. Nucleic Acids Res. 2017; 45:10229–10241. 28973446 10.1093/nar/gkx647 PMC5622339

[82] Tichon A., Gil N., Lubelsky Y., Havkin Solomon T., Lemze D., Itzkovitz S., Stern-Ginossar N., Ulitsky I. A conserved abundant cytoplasmic long noncoding RNA modulates repression by Pumilio proteins in human cells. Nat. Commun. 2016; 7:12209. 27406171 10.1038/ncomms12209 PMC4947167

[83] An J., Ponthier C.M., Sack R., Seebacher J., Stadler M.B., Donovan K.A., Fischer E.S. pSILAC mass spectrometry reveals ZFP91 as IMiD-dependent substrate of the CRL4CRBN ubiquitin ligase. Nat. Commun. 2017; 8:15398. 28530236 10.1038/ncomms15398 PMC5458144

[84] Conaway E.A., de Oliveira D.C., McInnis C.M., Snapper S.B., Horwitz B.H. Inhibition of inflammatory gene transcription by IL-10 is associated with rapid suppression of Lipopolysaccharide-Induced enhancer activation. J. Immunol. 2017; 198:2906–2915. 28213503 10.4049/jimmunol.1601781 PMC5369026

[85] Lau-Corona D., Suvorov A., Waxman D.J. Feminization of male mouse liver by persistent growth hormone stimulation: Activation of sex-biased transcriptional networks and dynamic changes in chromatin states. Mol. Cell Biol. 2017; 37:e00301-17. 28694329 10.1128/MCB.00301-17 PMC5599723

[86] Atger F., Gobet C., Marquis J., Martin E., Wang J., Weger B., Lefebvre G., Descombes P., Naef F., Gachon F. Circadian and feeding rhythms differentially affect rhythmic mRNA transcription and translation in mouse liver. Proc. Natl. Acad. Sci. U.S.A. 2015; 112:E6579–E6588. 26554015 10.1073/pnas.1515308112 PMC4664316

[87] Daum J.M., Keles O., Holwerda S.J., Kohler H., Rijli F.M., Stadler M., Roska B. The formation of the light-sensing compartment of cone photoreceptors coincides with a transcriptional switch. Elife. 2017; 6:e31437. 29106373 10.7554/eLife.31437 PMC5685475

[88] Feng H., Lopez G.Y., Kim C.K., Alvarez A., Duncan C.G., Nishikawa R., Nagane M., Su A.J., Auron P.E., Hedberg M.L. et al. EGFR phosphorylation of DCBLD2 recruits TRAF6 and stimulates AKT-promoted tumorigenesis. J. Clin. Invest. 2014; 124:3741–3756. 25061874 10.1172/JCI73093 PMC4151226

[89] Anastasi S., Fiorentino L., Fiorini M., Fraioli R., Sala G., Castellani L., Alema S., Alimandi M., Segatto O. Feedback inhibition by RALT controls signal output by the ErbB network. Oncogene. 2003; 22:4221–4234. 12833145 10.1038/sj.onc.1206516

[90] Sun T., Aceto N., Meerbrey K.L., Kessler J.D., Zhou C., Migliaccio I., Nguyen D.X., Pavlova N.N., Botero M., Huang J. et al. Activation of multiple proto-oncogenic tyrosine kinases in breast cancer via loss of the PTPN12 phosphatase. Cell. 2011; 144:703–718. 21376233 10.1016/j.cell.2011.02.003 PMC6014607

[91] Bracken C.P., Scott H.S., Goodall G.J. A network-biology perspective of microRNA function and dysfunction in cancer. Nat. Rev. Genet. 2016; 17:719–732. 27795564 10.1038/nrg.2016.134

[92] Gerstein M.B., Kundaje A., Hariharan M., Landt S.G., Yan K.K., Cheng C., Mu X.J., Khurana E., Rozowsky J., Alexander R. et al. Architecture of the human regulatory network derived from ENCODE data. Nature. 2012; 489:91–100. 22955619 10.1038/nature11245 PMC4154057

[93] Su W.L., Kleinhanz R.R., Schadt E.E. Characterizing the role of miRNAs within gene regulatory networks using integrative genomics techniques. Mol. Syst. Biol. 2011; 7:490. 21613979 10.1038/msb.2011.23 PMC3130556

[94] Greenberg M.E., Ziff E.B. Stimulation of 3T3 cells induces transcription of the c-fos proto-oncogene. Nature. 1984; 311:433–438. 6090941 10.1038/311433a0

[95] Kruijer W., Cooper J.A., Hunter T., Verma I.M. Platelet-derived growth factor induces rapid but transient expression of the c-fos gene and protein. Nature. 1984; 312:711–716. 6514007 10.1038/312711a0

[96] Muller R., Bravo R., Burckhardt J., Curran T. Induction of c-fos gene and protein by growth factors precedes activation of c-myc. Nature. 1984; 312:716–720. 6334806 10.1038/312716a0

[97] Greenberg M.E., Greene L.A., Ziff E.B. Nerve growth factor and epidermal growth factor induce rapid transient changes in proto-oncogene transcription in PC12 cells. J. Biol. Chem. 1985; 260:14101–14110. 3877054

[98] Lau L.F., Nathans D. Expression of a set of growth-related immediate early genes in BALB/c 3T3 cells: coordinate regulation with c-fos or c-myc. Proc. Natl. Acad. Sci. U.S.A. 1987; 84:1182–1186. 3469660 10.1073/pnas.84.5.1182 PMC304390

[99] Lamph W.W., Wamsley P., Sassone-Corsi P., Verma I.M. Induction of proto-oncogene JUN/AP-1 by serum and TPA. Nature. 1988; 334:629–631. 2457172 10.1038/334629a0

[100] Jacobsen A., Silber J., Harinath G., Huse J.T., Schultz N., Sander C. Analysis of microRNA-target interactions across diverse cancer types. Nat. Struct. Mol. Biol. 2013; 20:1325–1332. 24096364 10.1038/nsmb.2678 PMC3982325

