# Supplementary material for: Extensive transcriptional responses are co-ordinated by microRNAs as revealed by Exon–Intron Split Analysis (EISA)
Source: Nucleic Acids Res. 2019 Aug 2;47(16):8606–19. doi: 10.1093/nar/gkz664 (PMC6895270; doi:10.1093/nar/gkz664)

SUPPLEMENTARY DATA, Pillman et al.

Supplementary Table 1: Publically available datasets used within study

Supplementary Table 2: Kolmogorov–Smirnov (K-S) test p-values

K-S test p-values were calculated for all pairwise comparisons in Figure 1d.

Supplementary Table 3: Down-sampling indicates characteristics of high-throughput sequencing datasets for their applicability to EISA

Data from 6 HMLE and 4 MesHMLE sequencing runs were mixed and down-sampled to various depths to indicate the number of genes for which EISA can determine transcriptional and post-transcriptional changes. The maximum and minimum proportions of exonic reads and total intronic read numbers from the highest and lowest depth sample is indicated.

Supplementary Figure 1: miR-200b inhibits EGF-stimulated AKT activation in SHEP cells

A) SHEP (neuroblastoma) cells were stimulated with EGF as Fig.2c in the presence or absence of the miR-200 family members miR-200a and miR-200b. miR-200b (but not miR-200a) is predicted to have the same target specificity as miR-200c (used in Figure 2 and throughout the paper). We also find this to be the case across numerous assays (not shown). Total and activated AKT and ERK are determined by western. B) ERK activation was monitored in SHEP cells that had been transfected with siRNAs targeting MEK1 and MEK2. The surprising observation in (a) that miR-200b has little effect on ERK activation (in SHEP cells) we postulate is due to ERK activity being de-coupled from the traditional MEK/ERK signalling pathway that is targeted by miR-200b/c. This is indicated by the strong activation of ERK by EGF in siMEK-transfected cells.

Supplementary Figure 2: miR-200 inhibition drives a transcriptional response in HMLE cells

A) mRNA expression (from RNA-Seq) after miR-200 inhibition in HMLE cells indicate an early / modest EMT response. B) EISA plot from RNA-seq data for HMLE cells with and without inhibition of miR-200c. Red dots represent the top 10% of genes that were most regulated upon miR-200 inhibition. The remaining 90% of genes are within the blacked out region. C) EISA was used to plot genes that are responsive to miR-200 inhibition on a ΔI: ΔE axis. Genes are coloured according to the strength of their direct targeting by miR-200 (as predicted by TargetScan context score). The position of strongly predicted targets is consistent with post-transcriptional de-repression after miR-200 inhibition. D) Log Fold change (total expression, top; post-transcriptional change, bottom) for each of the TFs of interest identified through miR-200c expression (Fig.3d). E) Enriched gene ontologies among genes that were both transcriptionally upregulated after miR-200c expression and transcriptionally downregulated after miR-200 inhibition identifies similar processes to those reported in Figure 3.

Supplementary Figure 3: miR-200 inhibition drives a transcriptional response in MDCK cells

A) mRNA expression (from RNA-Seq) after miR-200 inhibition in MDCK cells indicate an EMT response. B) Red dots represent individual genes that were among the top 10% that were most regulated upon miR-200 inhibition. The blacked out region represents the least changing 90% of genes. Spread along the x axis (ΔI) indicates a predominantly transcriptional response. C) EISA was used to plot genes that are responsive to miR-200 inhibition on a ΔI: ΔE axis. Genes are coloured according to the strength of their direct targeting by miR-200 (as predicted by TargetScan context score). D) Log Fold change (total expression, MDCK cells) for each of the TFs of interest identified through miR-200c expression in HMLE cells (Fig.3d). E) Heat map displaying the TFs that increased at least 2 fold after miR-200 inhibition in MDCK cells. The likelihood of direct targeting by miR-200 is indicated via TargetScan Context Score. F) Enriched gene ontologies among genes that were transcriptionally downregulated (left) and transcriptionally upregulated (right) after miR-200 inhibition in MDCKs.

Supplementary Figure 4: Epithelial/mesenchymal expression of miR-200 regulated TFs in breast cancer TCGA data

A) Pearson’s correlation was calculated from TCGA breast cancer data correlated with EMT signature scores (Foroutan et al, BMC Bioinformatics 2018). B-D) BNC2 expression is derived from breast cancer TCGA data, subdivided by PAM50 subtype (C,D).

Supplementary Figure 5: Extensive buffering responses occur between transcriptional and post-transcriptional gene regulatory layers

ΔE-ΔI (post-transcription, y axis) was graphed against ΔI (transcription, x axis) to represent EISA-defined gene regulatory effects. Relative contributions of the two gene regulatory arms are shown after miRNA-perturbation (from Fig 3 and 4). Genes displayed represent the best 200 (top left), best 500 (top right) or all targets (bottom left) predicted by TargetScan. Each of these were also targets predicted by DIANA microT-CDS. “No Targets” (bottom right) are genes identified by EISA, but are not predicted targets by either prediction algorithm. A line of best fit and R^2 value are indicated. Bottom histograms display the % number of genes that are either post-transcriptionally up- or down-regulated by ≥ 1.25 (left) or ≥ 2-fold (right). A bias toward post-transcriptional downregulation is anticipated for direct miRNA targets (but not for “No Targets”). This is prominent in almost all datasets, supporting the quality of the data being examined. miR-106a only showed a modest tendency toward the post-transcriptional downregulation of predicted targets, though this might reflect targeting specificities beyond seed-dependency.

Supplementary Table 1

Comparison	Sample	ENA study num.	ENA sample num.	ENA sample name
HMLE vs MesHMLE	H_r3	PRJEB25061	ERS2212956	HMLE_polyAminus_rep3
HMLE vs MesHMLE	H_r4	PRJEB8225	ERS640277	HMLE_polyAminus_rep2
HMLE vs MesHMLE	Mneg_r1	PRJEB25061	ERS2212957	MesHMLE_sineg_polyAminus_rep1
HMLE vs MesHMLE	Mneg_r2	PRJEB25061	ERS2212958	MesHMLE_sineg_polyAminus_rep2
HMLE vs MesHMLE	M_r3	PRJEB8225	ERS640279	mesHMLE_polyAminus_rep2
------	------	------	------	------
MesHMLE vs MesHMLE+ miR200c	M_r3	PRJEB25042	ERS2210893	mesHMLE_polyAplus_rep1
MesHMLE vs MesHMLE+ miR200c	Mneg_r1	PRJEB25042	ERS2210894	mesHMLE_polyAplus_rep2
MesHMLE vs MesHMLE+ miR200c	Mneg_r2	PRJEB25042	ERS2210895	mesHMLE_polyAplus_rep3
MesHMLE vs MesHMLE+ miR200c	200c_r1	PRJEB25042	ERS2210898	mesHMLE+miR-200c_polyAplus_rep1
MesHMLE vs MesHMLE+ miR200c	200c_r2	PRJEB25042	ERS2210899	mesHMLE+miR-200c_polyAplus_rep2

Studied miRNA/protein	Repository	Accession
ZEB1	Gene Expression Omnibus	GSE81167
miRNA-1343	Gene Expression Omnibus	GSE75591
miRNA-106a	Gene Expression Omnibus	GSE62678
miRNA-137	Gene Expression Omnibus	GSE53220
miRNA-494	Gene Expression Omnibus	GSE64615
miRNA-155	Gene Expression Omnibus	GSE21992
miRNA-155; miRNA-124	Gene Expression Omnibus	GSE52530
miRNA-372	Gene Expression Omnibus	GSE81417

Supplementary Table 2

Kolmogorov–Smirnov (K-S) test p-values for Figure 1d							
dExon							
	no site	6mer	7mer	8mer	> 1 site	> 2 sites	> 3 sites
no site	1	4.96E-05	2.84E-08	2.89E-09	5.65E-12	4.86E-11	2.90E-07
6mer	4.96E-05	1	0.003611	4.65E-05	8.36E-09	7.22E-09	1.13E-05
7mer	2.84E-08	0.003611	1	0.012194	0.002618	5.48E-06	0.000212
8mer	2.89E-09	4.65E-05	0.012194	1	0.531732	0.156222	0.05289
> 1 site	5.65E-12	8.36E-09	0.002618	0.531732	1	0.058272	0.031147
> 2 sites	4.86E-11	7.22E-09	5.48E-06	0.156222	0.058272	1	0.869553
> 3 sites	2.90E-07	1.13E-05	0.000212	0.05289	0.031147	0.869553	1
dExon-Intron							
	no site	6mer	7mer	8mer	> 1 site	> 2 sites	> 3 sites
no site	1	6.32E-09	2.49E-12	3.84E-16	3.44E-18	4.81E-18	8.72E-14
6mer	6.32E-09	1	0.010341	9.97E-07	1.59E-08	9.33E-09	5.91E-07
7mer	2.49E-12	0.010341	1	0.000936	0.001888	3.85E-05	2.95E-05
8mer	3.84E-16	9.97E-07	0.000936	1	0.82919	0.470493	0.023249
> 1 site	3.44E-18	1.59E-08	0.001888	0.82919	1	0.163753	0.011721
> 2 sites	4.81E-18	9.33E-09	3.85E-05	0.470493	0.163753	1	0.222386
> 3 sites	8.72E-14	5.91E-07	2.95E-05	0.023249	0.011721	0.222386	1
dIntron							
	no site	6mer	7mer	8mer	> 1 site	> 2 sites	> 3 sites
no site	1	0.344216	0.434118	0.955709	0.996912	0.282563	0.788513
6mer	0.344216	1	0.989748	0.957267	0.57188	0.202269	0.810568
7mer	0.434118	0.989748	1	0.934607	0.707367	0.128114	0.685433
8mer	0.955709	0.957267	0.934607	1	0.999282	0.404585	0.878528
> 1 site	0.996912	0.57188	0.707367	0.999282	1	0.369388	0.890632
> 2 sites	0.282563	0.202269	0.128114	0.404585	0.369388	1	0.995656
> 3 sites	0.788513	0.810568	0.685433	0.878528	0.890632	0.995656	1

Supplementary Table 3

Depth (million reads)	# genes called by EISA	% of exon reads (max)	% of exon reads (min)	Intron reads (max)	Intron reads (min)
60	8155	95	89	4739802	1957160
50	7935	95	89	3936278	1625256
40	7630	95	89	3134354	1292555
30	7291	95	88	2327478	959996
20	6581	95	88	1520922	624514
10	4906	95	87	709984	285165
5	2852	95	85	290622	113153

Supplementary Figure 1

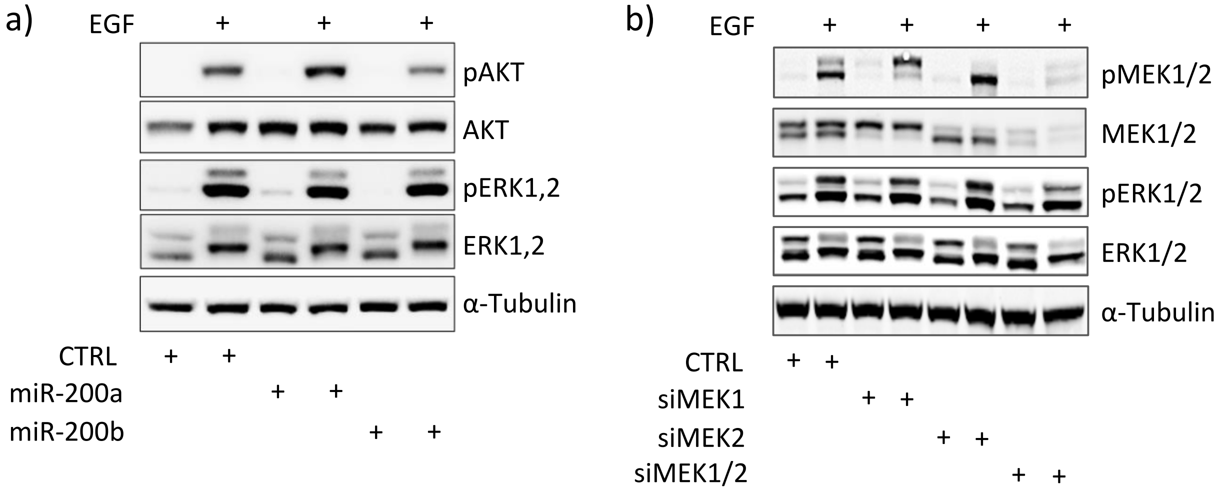

Supplementary Figure 2

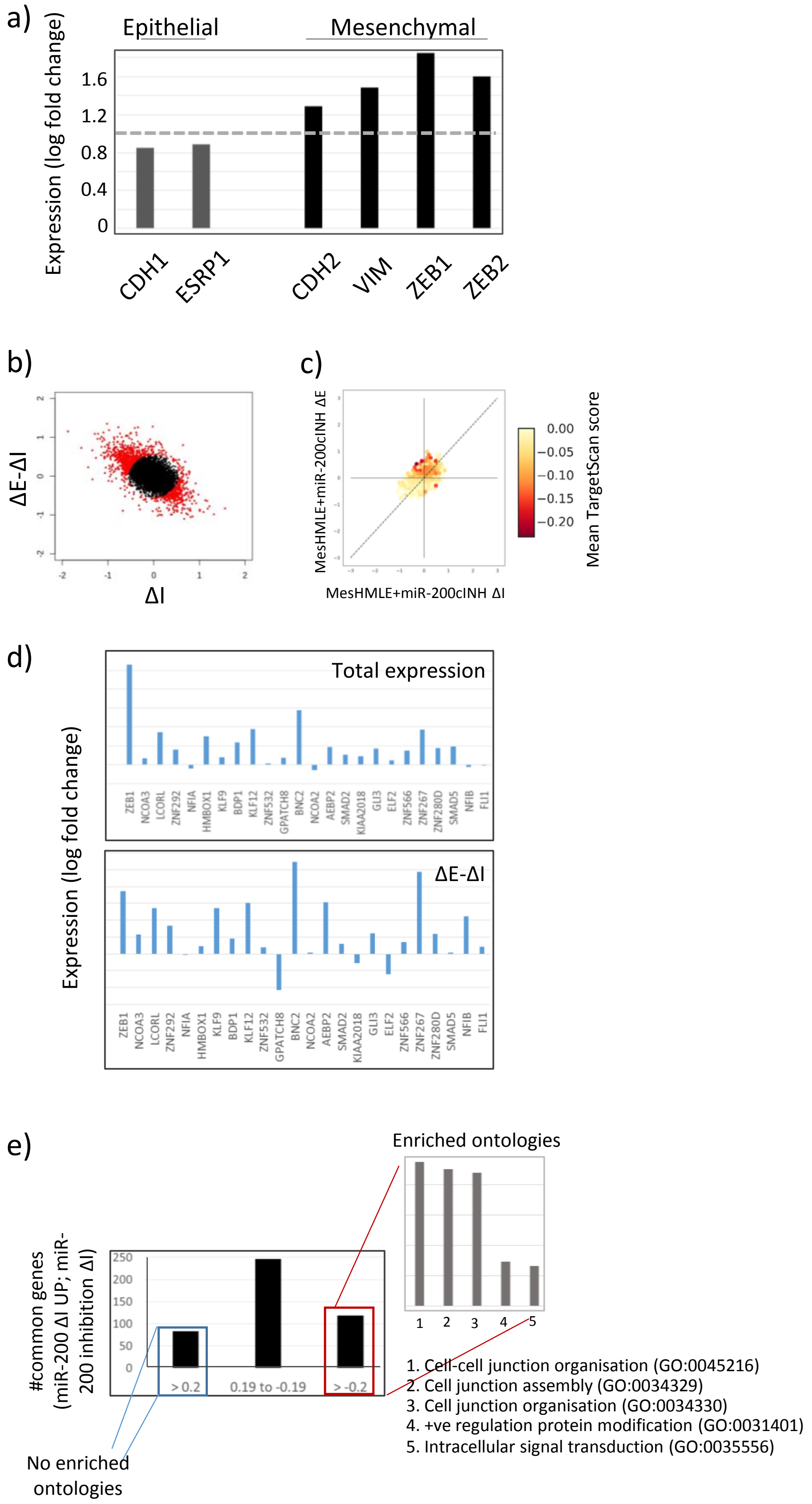

Supplementary Figure 3

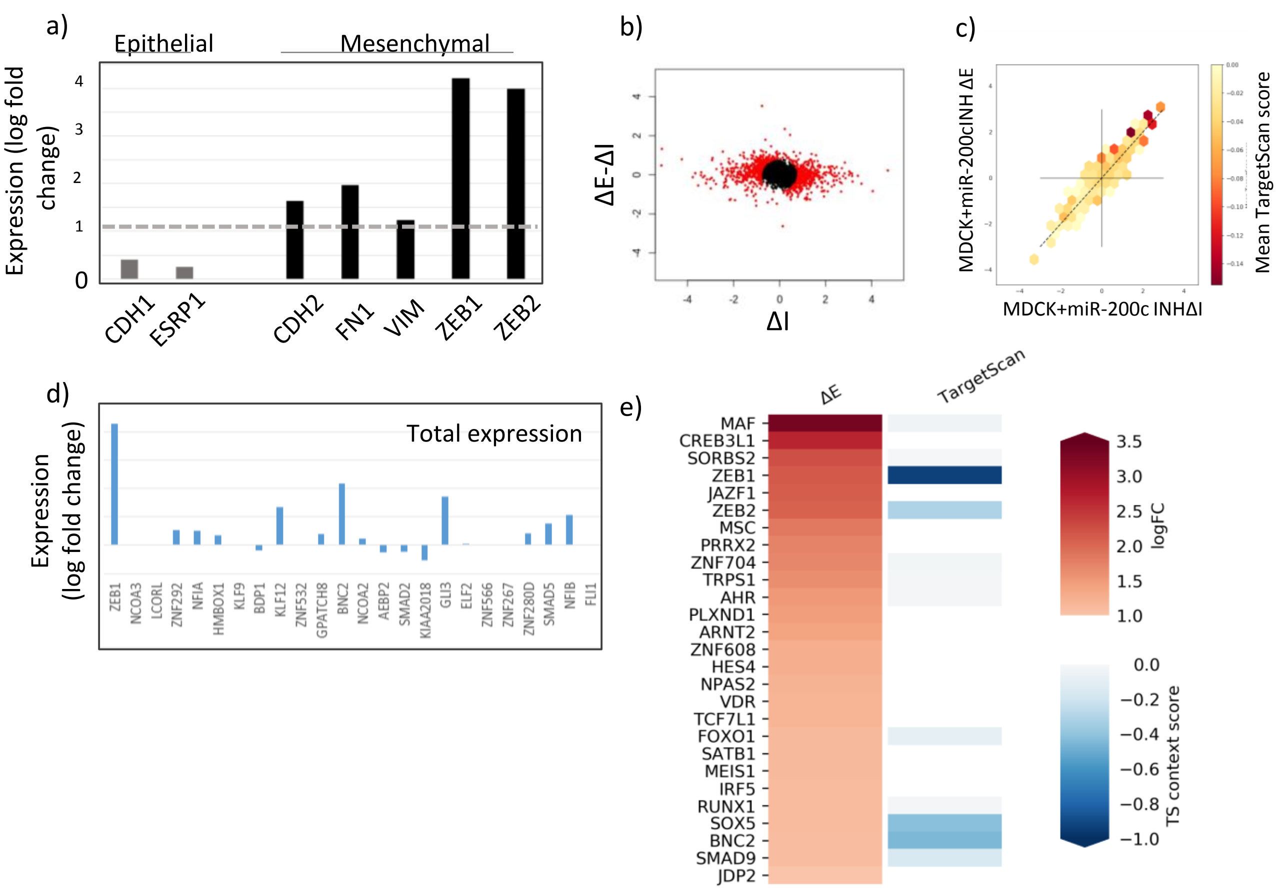

f)

Ontology	Enrichment
basement membrane organization (GO:0071711)	13.29
embryonic placenta development (GO:0001892)	5.5
cornification (GO:0070268)	4.7
cell junction assembly (GO:0034329)	4.06
cell junction organization (GO:0034330)	3.4
regulation of epithelial cell migration (GO:0010632)	3.35
angiogenesis (GO:0001525)	3.04
morphogenesis of an epithelium (GO:0002009)	2.61
blood vessel morphogenesis (GO:0048514)	2.6
tube morphogenesis (GO:0035239)	2.6
epidermis development (GO:0008544)	2.55
actin cytoskeleton organization (GO:0030036)	2.48
blood vessel development (GO:0001568)	2.46
epithelial cell differentiation (GO:0030855)	2.42

Ontology	Enrichment
collagen fibril organization (GO:0030199)	10.17
regulation of cartilage development (GO:0061035)	8.38
regulation of sprouting angiogenesis (GO:1903670)	6.98
positive regulation of endothelial cell migration (GO:0010595)	6.75
regulation of cytokine production in immune response (GO:0002718)	6.6
regulation of epithelial to mesenchymal transition (GO:0010717)	6.2
chondrocyte differentiation (GO:0002062)	6.05
regulation of TGF-beta receptor signaling pathway (GO:0017015)	5.9
platelet degranulation (GO:0002576)	5.85
regulation of cellular response to TGF-beta stimulus (GO:1903844)	5.79
regulation of blood vessel endothelial cell migration (GO:0043535)	5.78
negative regulation of response to growth factor stimulus (GO:0090288)	5.77
positive regulation of epithelial cell migration (GO:0010634)	5.72
regulation of endothelial cell migration (GO:0010594)	5.5
postsynapse organization (GO:0099173)	5.44
negative regulation of cell migration (GO:0030336)	5.32
roof of mouth development (GO:0060021)	5.25
regulation of cellular response to growth factor stimulus (GO:0090287)	5.2
regulation of epithelial cell migration (GO:0010632)	5.03

Supplementary Figure 4

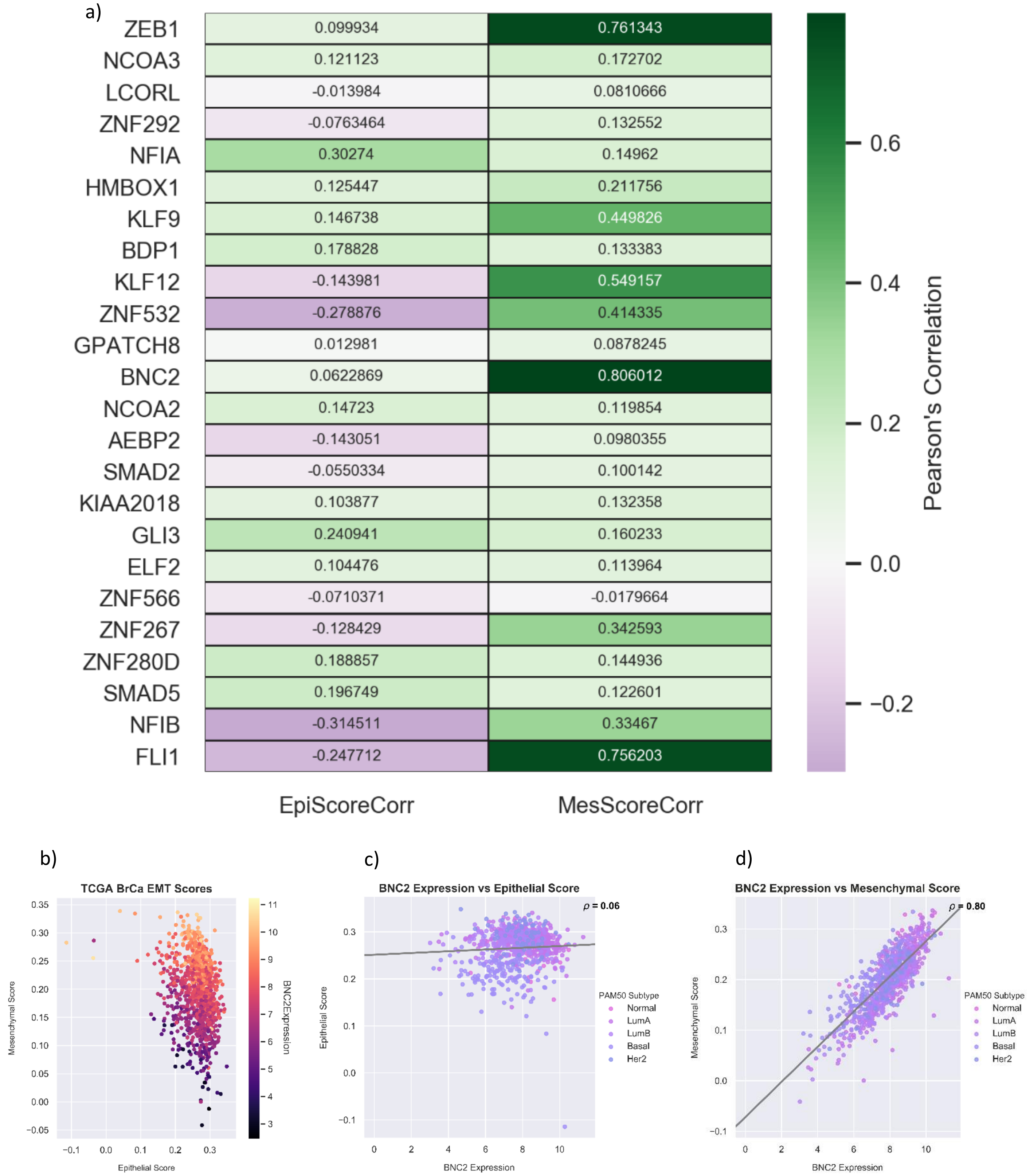

Supplementary Figure 5 (i)

miR-200c

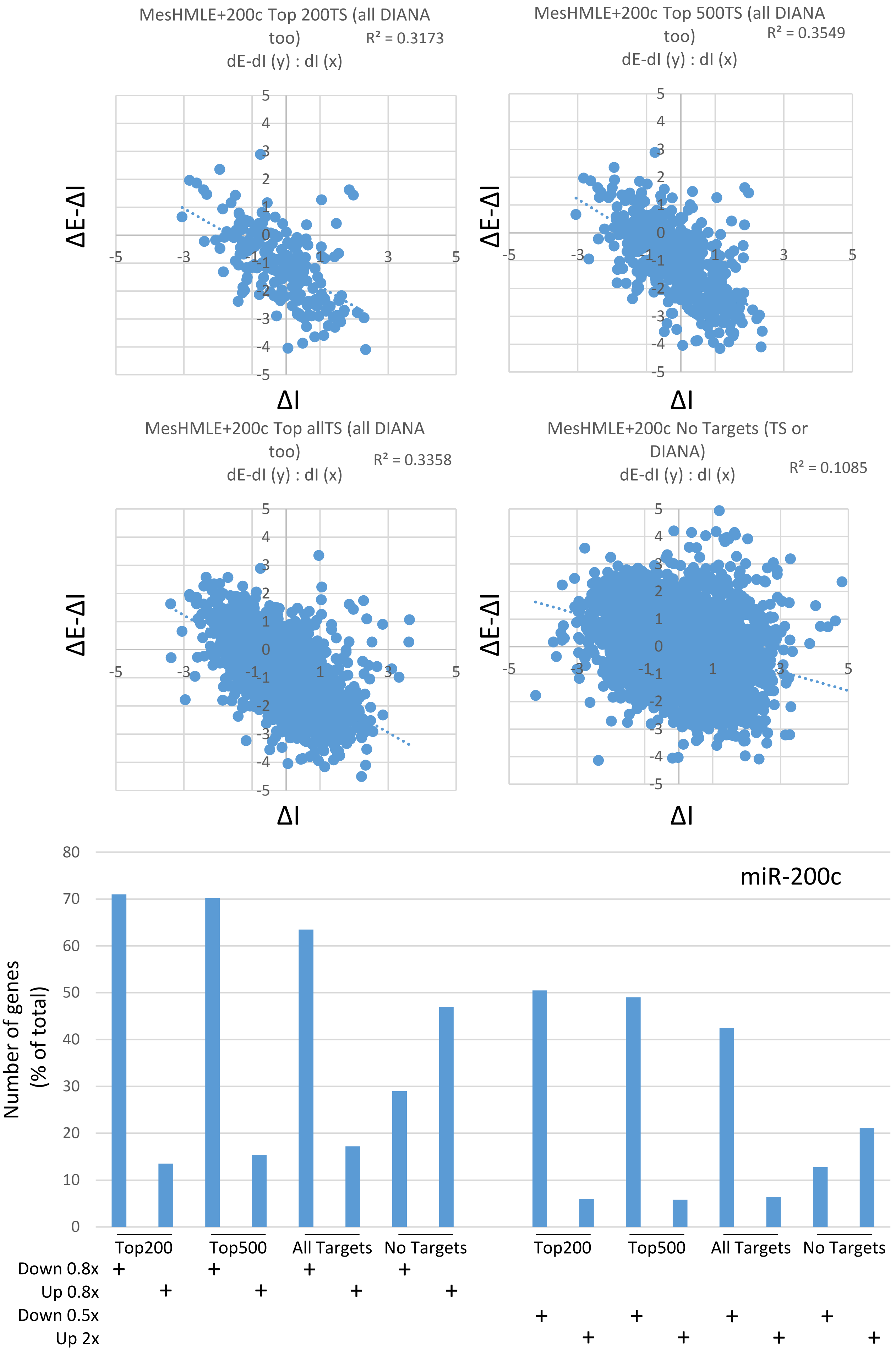

Supplementary Figure 5 (ii)

miR-106a

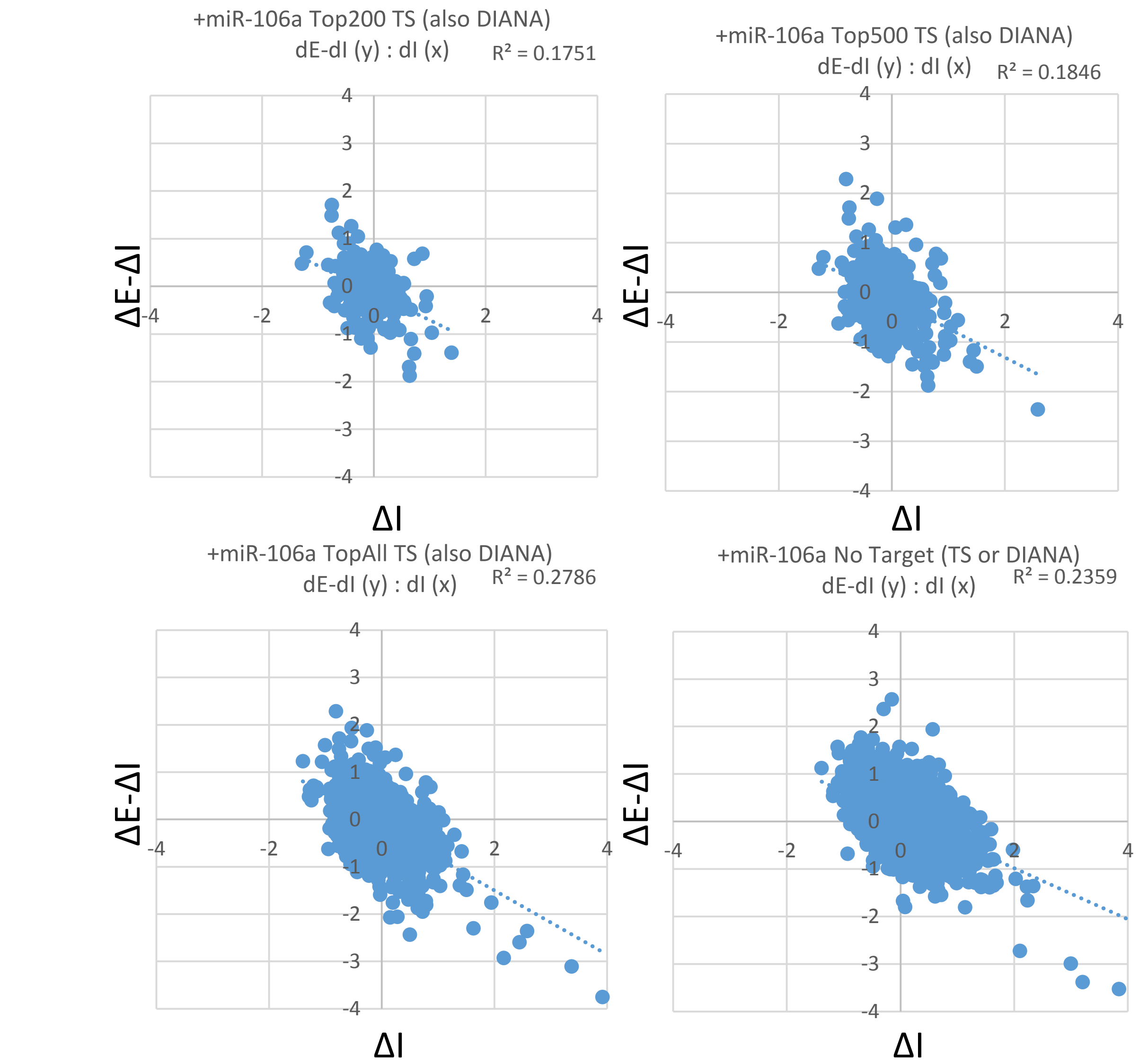

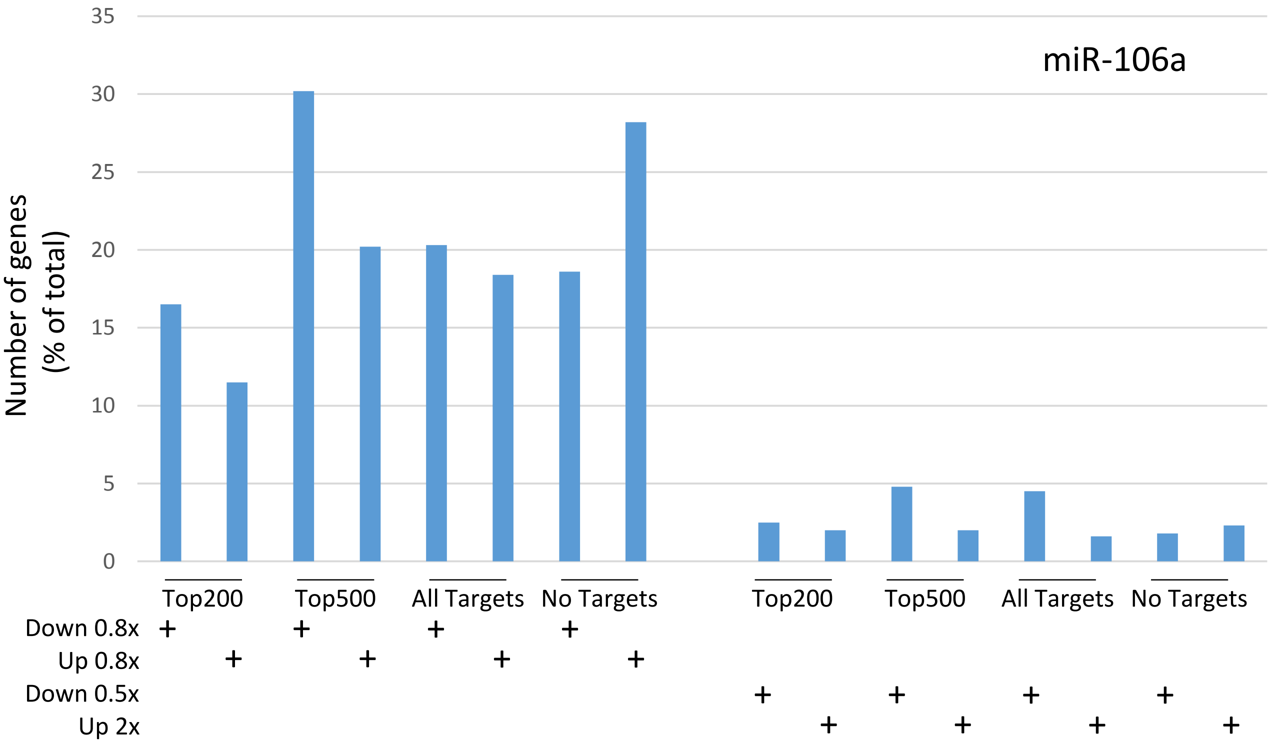

Supplementary Figure 5 (iii)

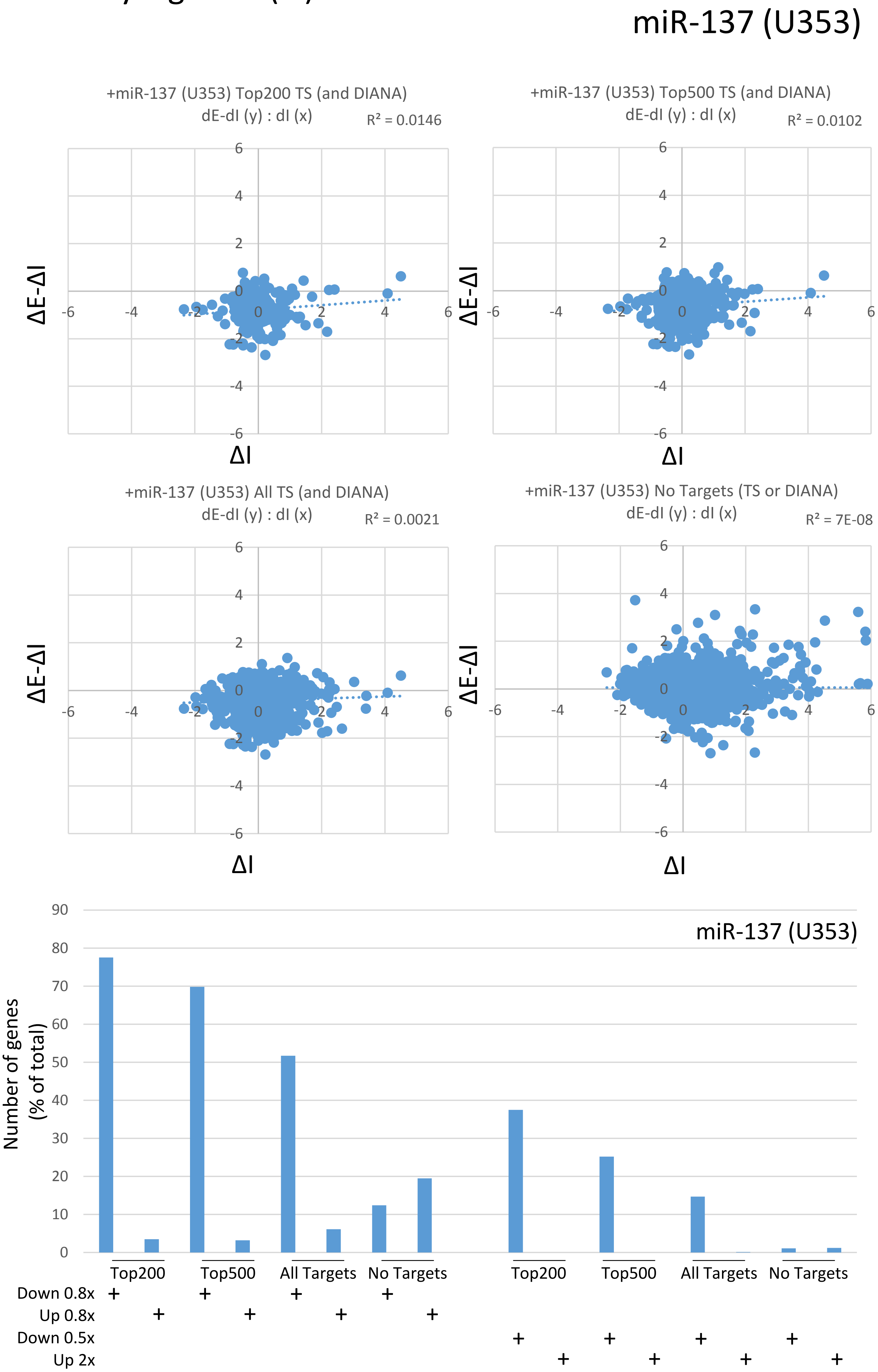

Supplementary Figure 5 (iv)

miR-137 (U251)

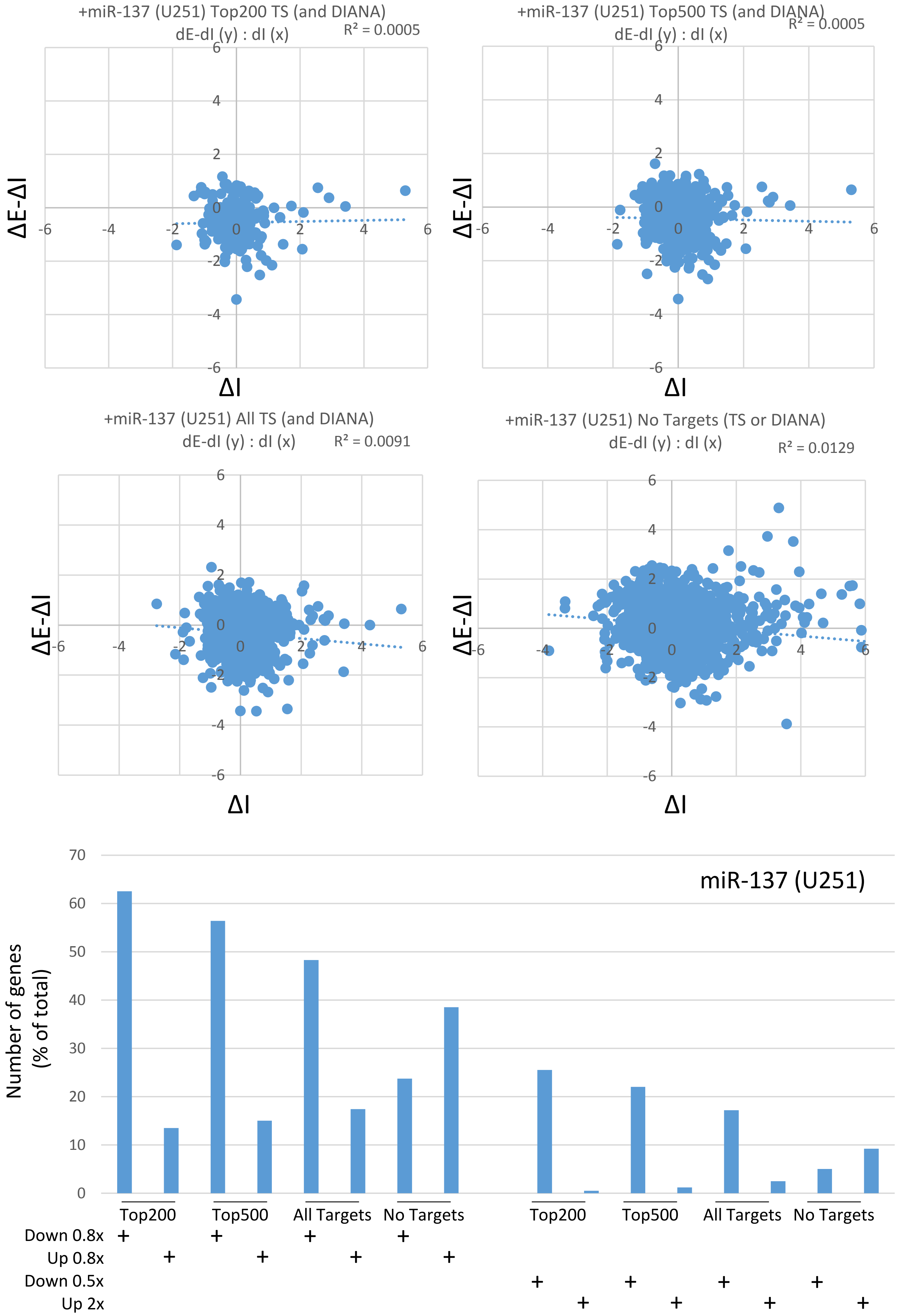

Supplementary Figure 5 (v)

miR-372-3p

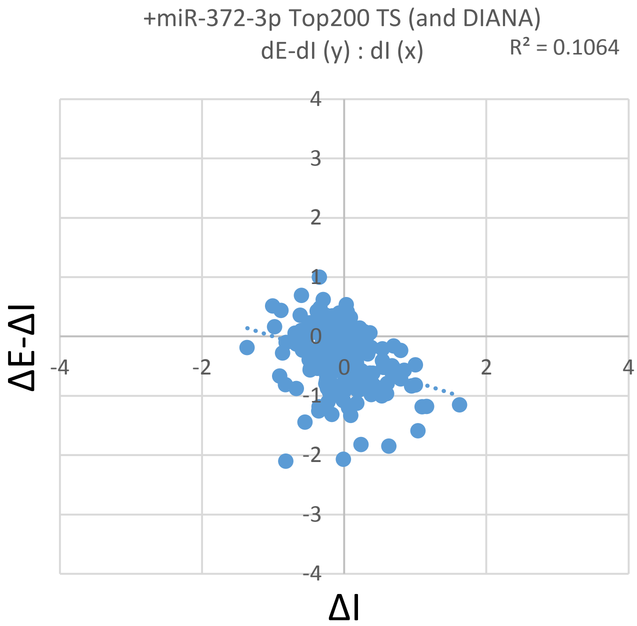

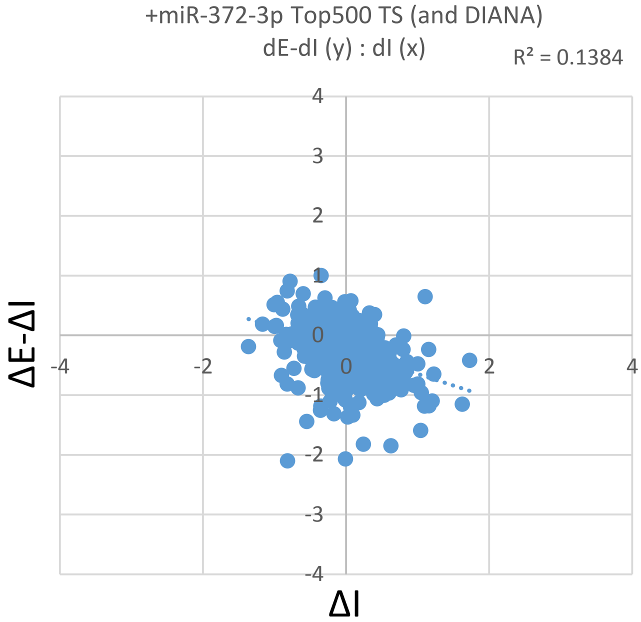

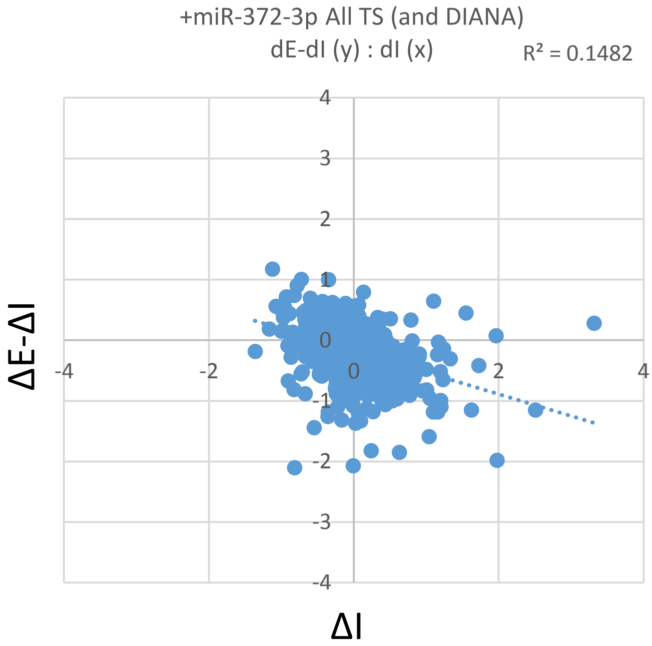

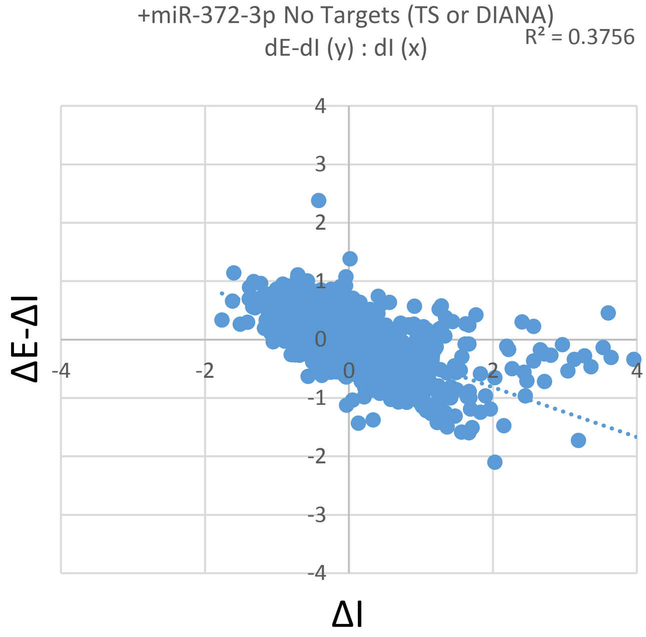

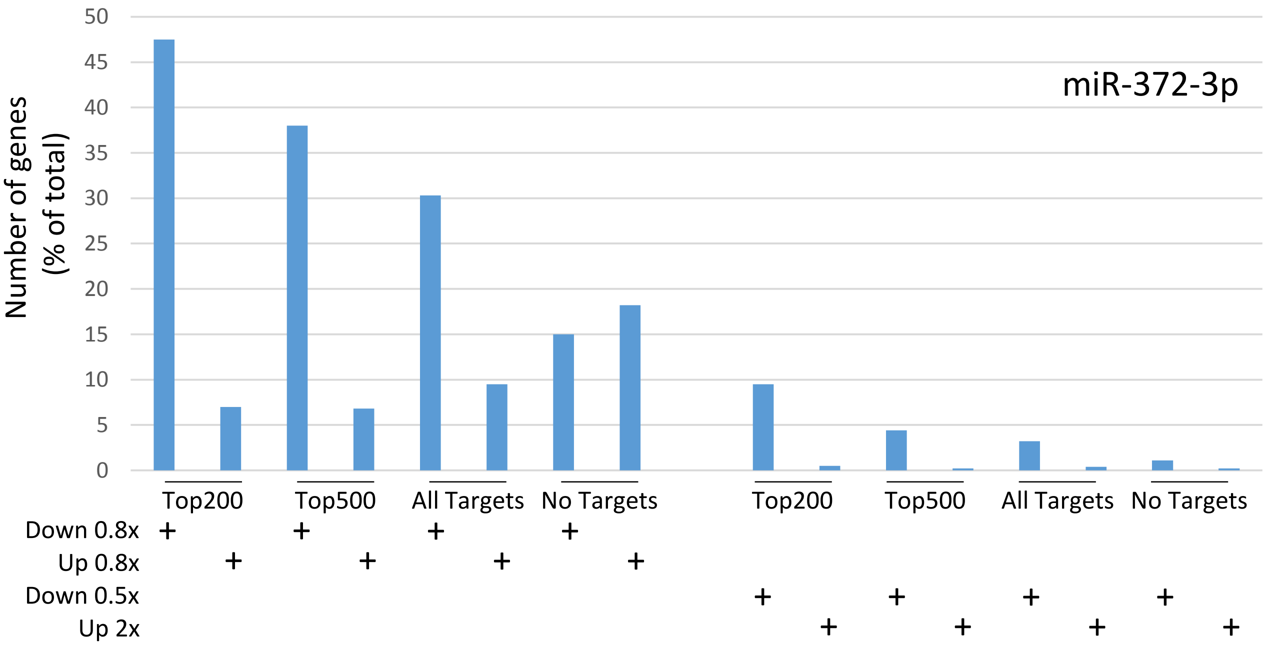

Supplementary Figure 5 (vi)

miR-494

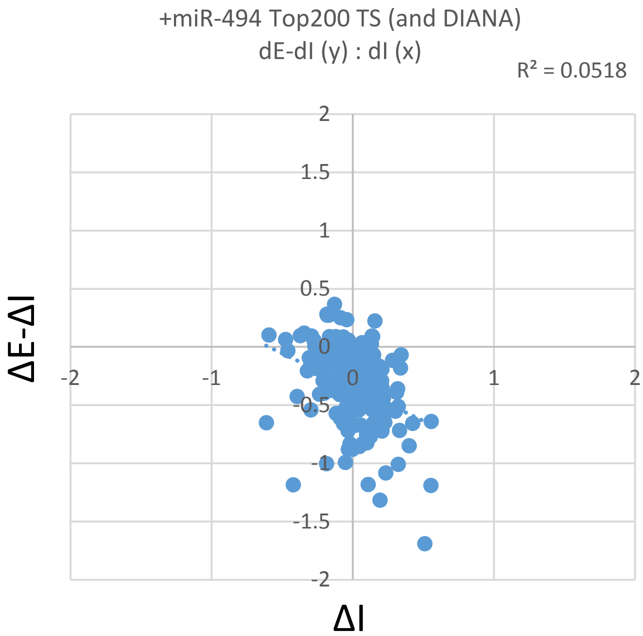

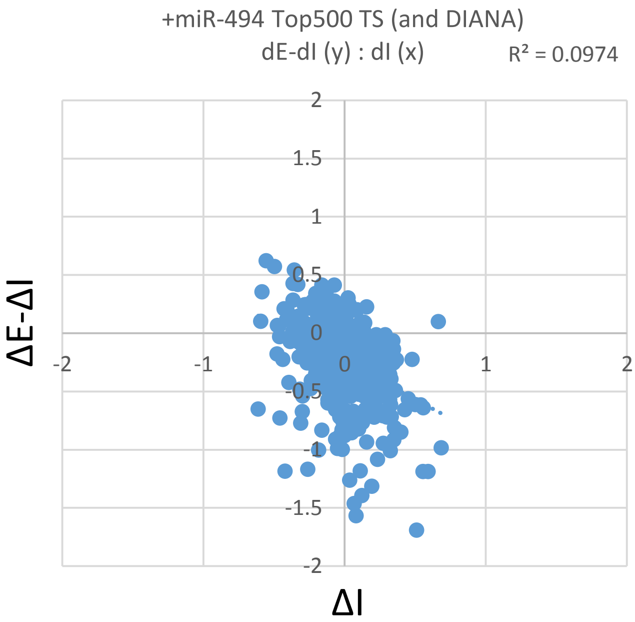

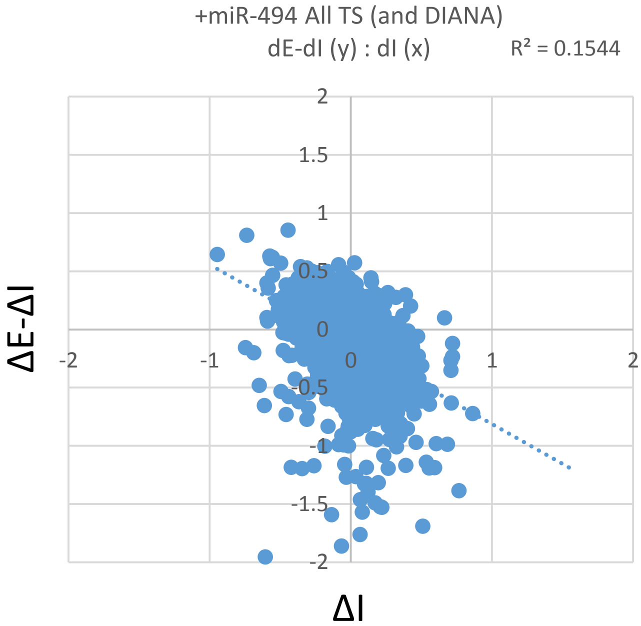

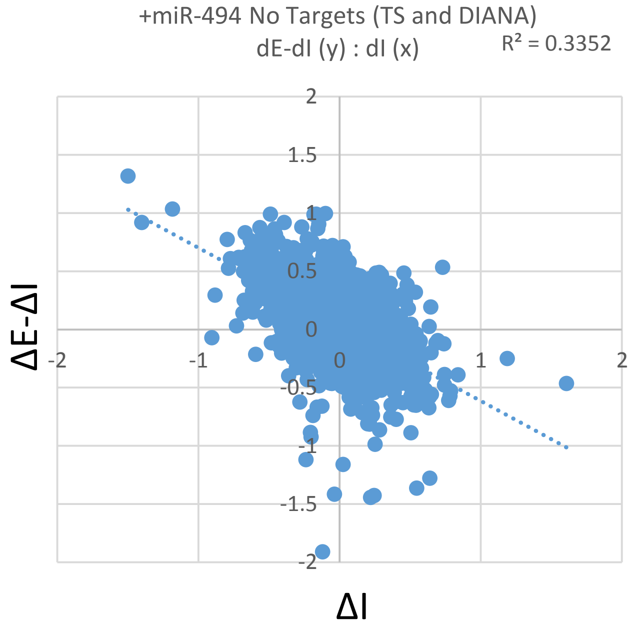

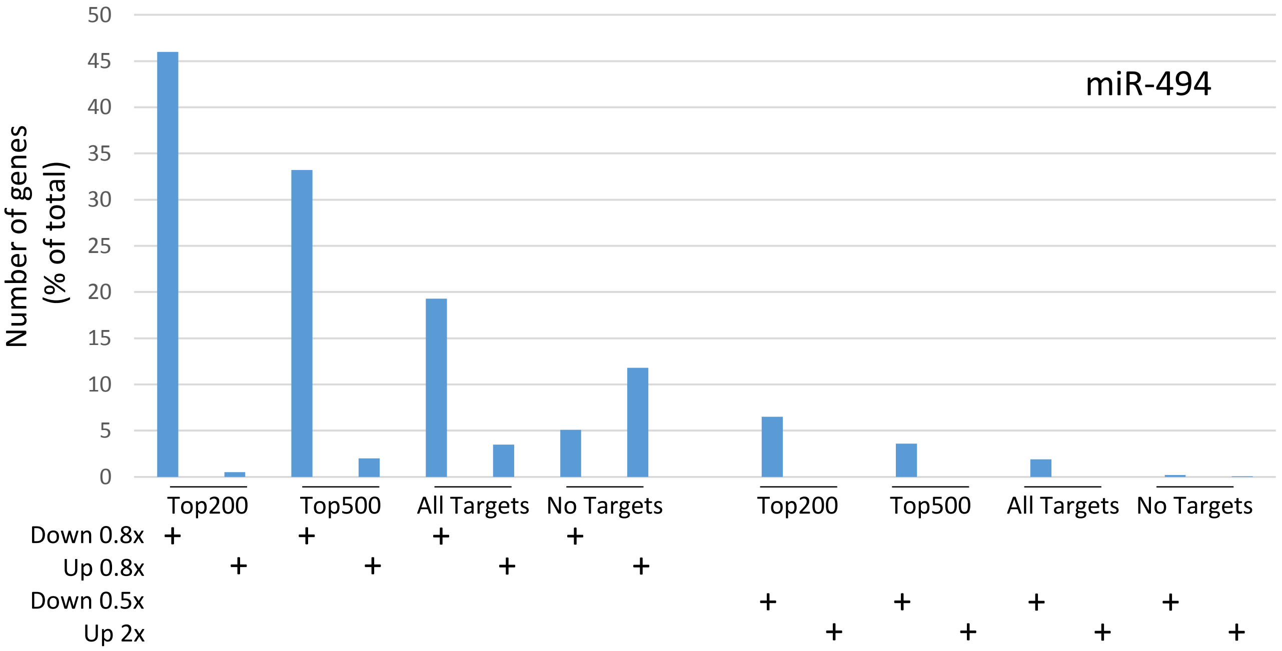

Supplementary Figure 5 (vii)

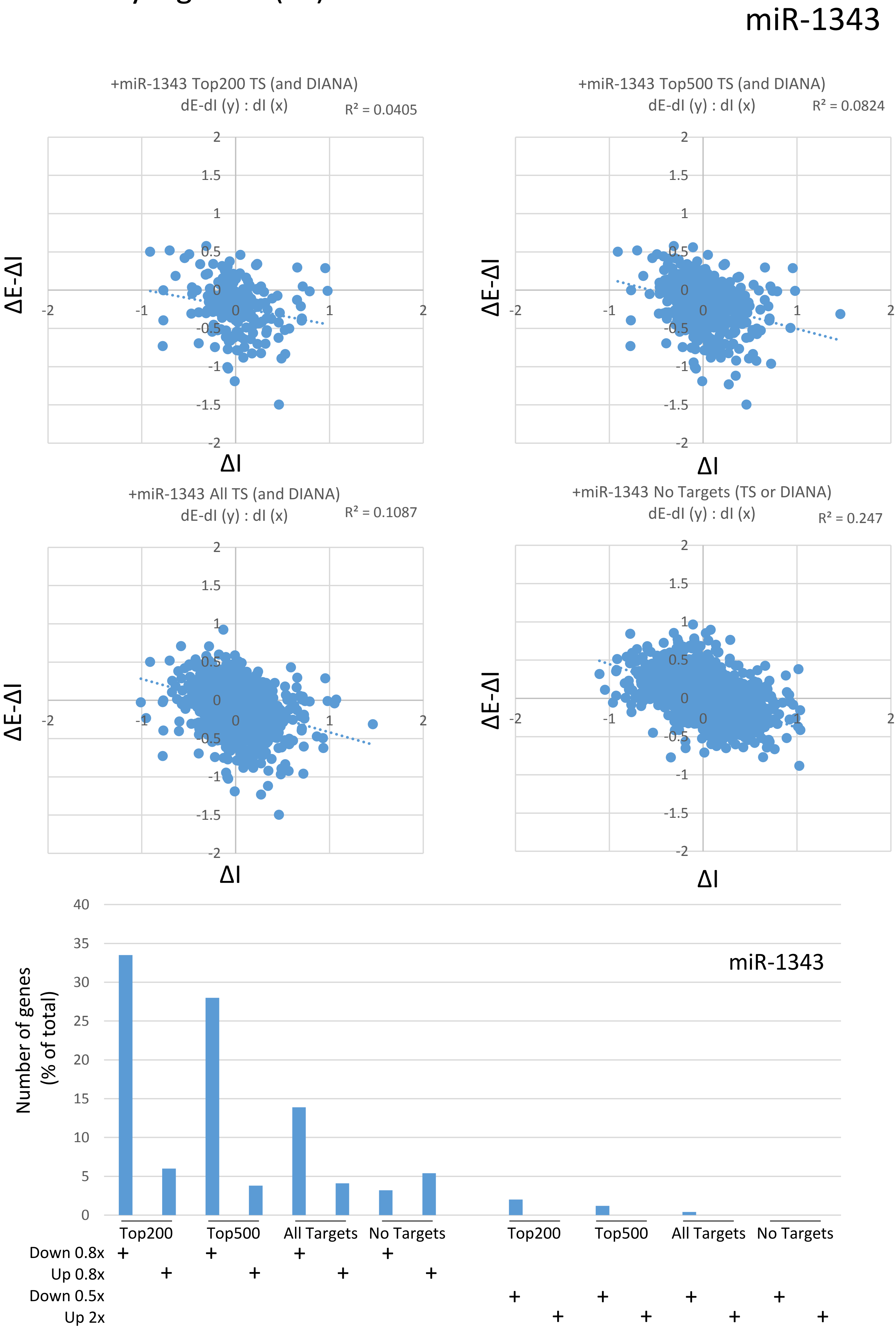

Supplementary Figure 5 (viii)

miR-124

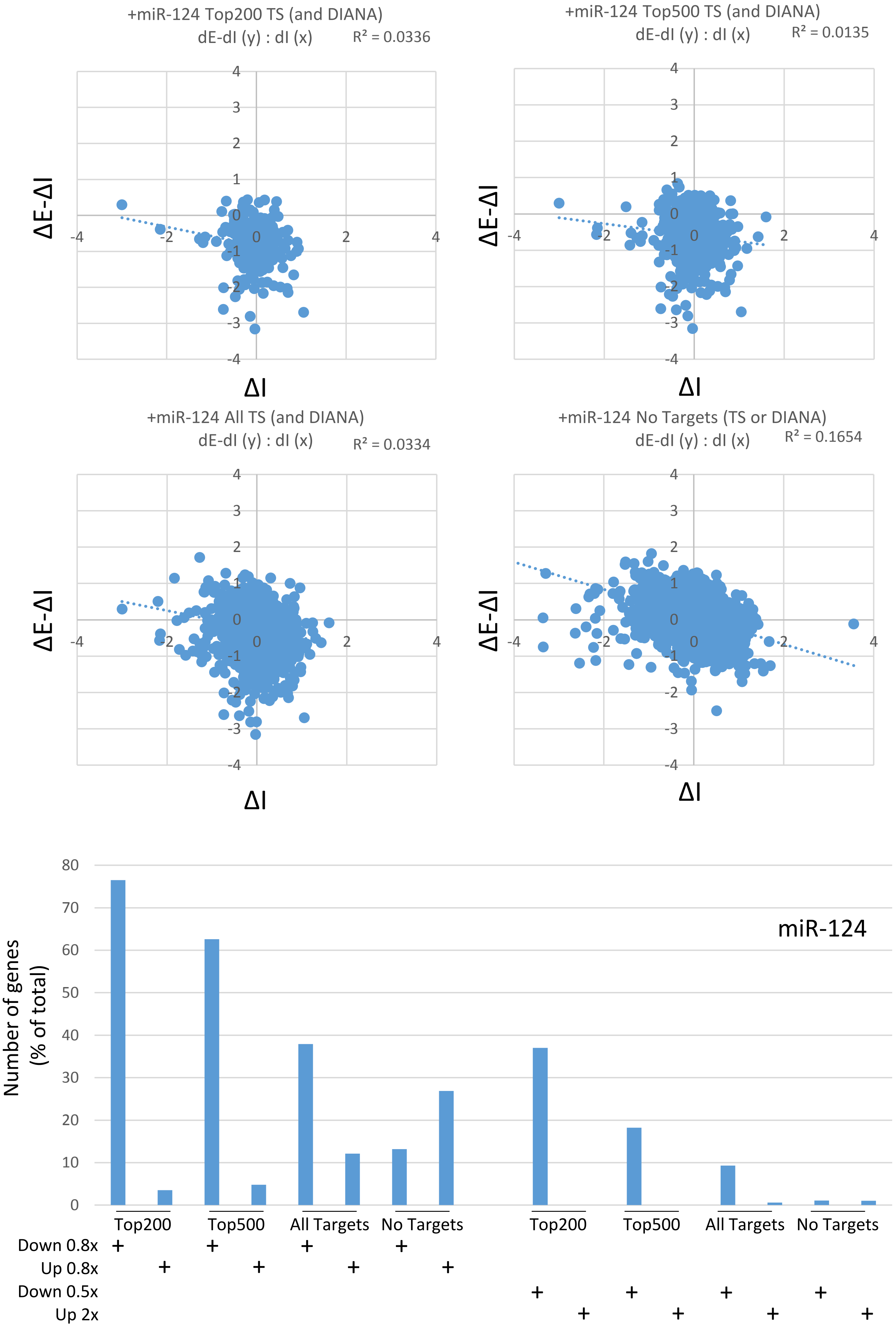

Supplementary Figure 5 (ix)

miR-155

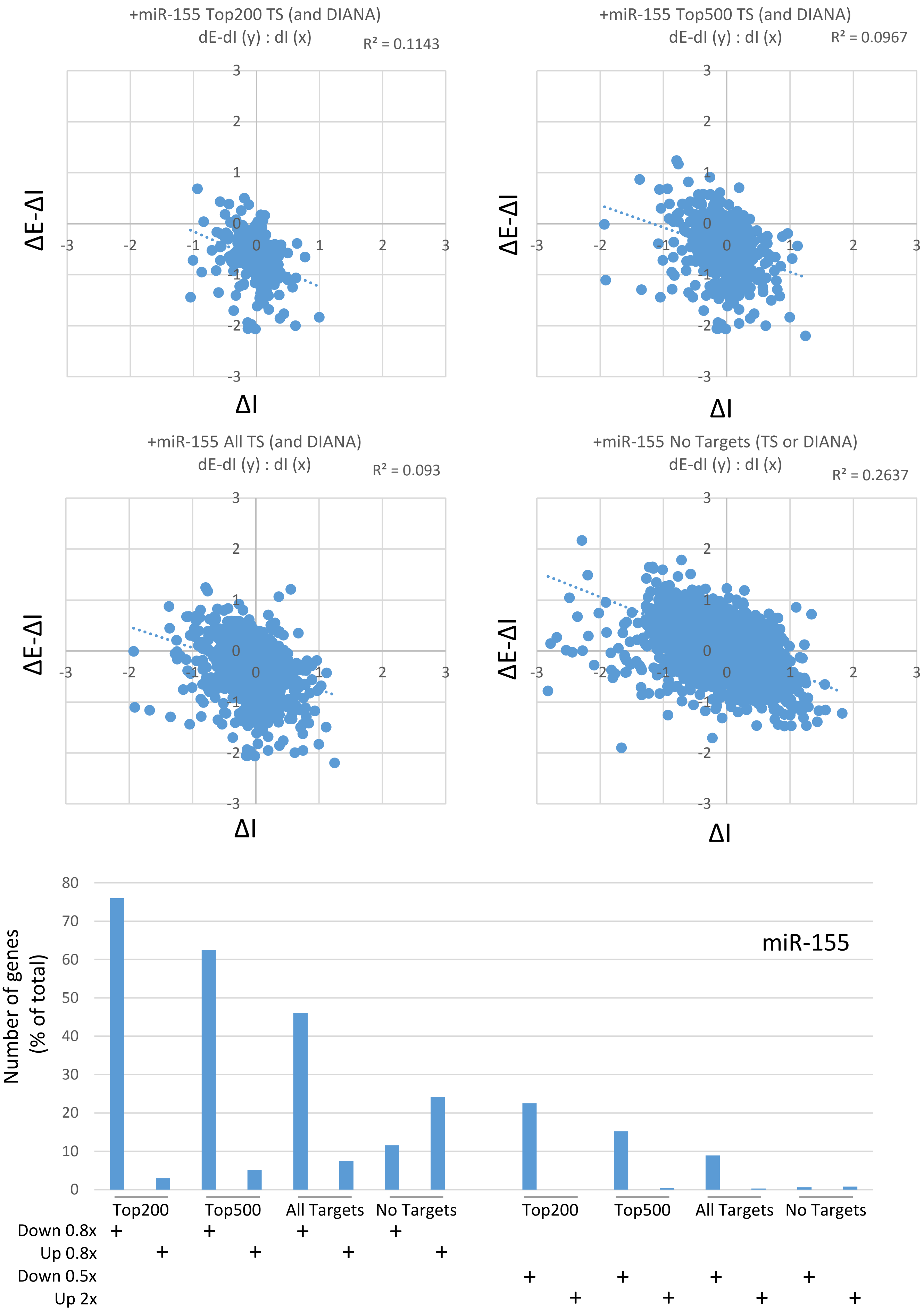

Supplement: gkz664_Supplemental_Files [file gkz664_supplemental_files.pdf]
